# Tumor Microenvironment-on-a-Chip: Construction and Application in Traditional Chinese Medicine Anti-Tumor Therapy

**DOI:** 10.3390/bios16080413

**Published:** 2026-07-30

**Authors:** Yujie Sheng, Wei Chen, Ziyi Zhang, Ziyi Cui, Peiju Zhong, Yu Xia, Zihan Yang

**Affiliations:** 1 College of Basic Medical Sciences, Hubei University of Chinese Medicine, Wuhan 430065, China; 2 Hubei Shizhen Laboratory, Wuhan 430061, China; 3 Department of Gastroenterology and Hepatology, Tongji Hospital, Tongji Medical College, Huazhong University of Science and Technology, Wuhan 430030, China; 4 School of Life Sciences, The Chinese University of Hong Kong, Shatin, New Territories, Hong Kong 999077, China

**Keywords:** microfluidic, tumor microenvironment, traditional Chinese medicine, biotechnology, biomedical applications

## Abstract

Cancer is the second leading cause of death worldwide, and tumor heterogeneity remains a major obstacle to effective therapy. In vitro reconstruction of the tumor microenvironment (TME) is particularly challenging because of its complexity, dynamic nature, and spatial heterogeneity, which limits the predictive value of conventional models for anticancer drug evaluation. Traditional Chinese medicine (TCM) has attracted increasing attention in cancer therapy owing to its multi-component, multi-target, and multi-pathway therapeutic characteristics. However, the complexity of TCM formulations and the diversity of their bioactive constituents make their pharmacological mechanisms difficult to elucidate using conventional experimental models. Microfluidic tumor microenvironment-on-a-chip (TME-on-a-chip) platforms integrate engineered cell culture with dynamic perfusion systems to recapitulate key structural, biochemical, and cellular features of the TME, thus providing a more physiologically relevant platform for anticancer research. With advantages such as low sample consumption, precise spatiotemporal control, multicellular co-culture, and real-time monitoring, these platforms provide a promising strategy for evaluating the efficacy and microenvironment-dependent effects of TCM-derived compounds and formulations. In this review, we summarize recent advances in the construction of TME-on-a-chip models, including multicellular organization, extracellular matrix simulation, vascularization, and immune microenvironment reconstruction, and discuss how these systems can be applied to key questions in TCM-based anticancer research. We further analyze current technical and methodological challenges that limit their broader adoption in TCM research and highlight future directions for promoting mechanism-driven and precision-oriented development of TCM in cancer therapy.

## 1. Introduction

Cancer remains one of the most serious threats to human health worldwide, with more than 20 million new cancer cases and nearly 10 million deaths reported globally in 2022. Lung cancer, colorectal cancer, liver cancer, breast cancer, and stomach cancer are among the major contributors to cancer-related mortality [1]. Current cancer treatment methods primarily include surgical resection, chemotherapy, radiotherapy, targeted therapy, and immunotherapy [2]. However, these approaches face multiple challenges; in recent years, their overall effectiveness is still limited. Many tumors present with subtle or nonspecific symptoms in the early stage, leading to delayed diagnosis and reduced chances for curative treatment. In addition, tumor heterogeneity leads to variability in therapeutic responses, while tumor invasion, metastasis, and the gradual development of drug resistance further complicate clinical management [3]. Against this backdrop, the tumor microenvironment (TME) has emerged as a critical factor influencing tumor progression and therapeutic outcomes. This complex ecosystem, composed of tumor cells, immune cells, fibroblasts, extracellular matrix, and various signaling molecules, directly regulates tumor progression and therapeutic failure through mechanisms like angiogenesis, immune evasion, and metabolic reprogramming which collectively shape tumor behavior and treatment response [4]. Increasing attention has therefore been directed toward understanding the role of the TME, as targeting this complex microenvironment may provide new opportunities for improving anticancer strategies and overcoming therapeutic resistance.

Traditional Chinese medicine (TCM), a holistic medical system developed over thousands of years, is increasingly recognized as a complementary strategy in cancer therapy. Unlike conventional single-target drugs, TCM emphasizes multi-component and multi-target regulation, which may exert anti-tumor effects through modulation of the TME [5]. Emerging studies suggest that tonic herbs such as Astragalus and Ginseng can enhance immune responses by activating T cells and Natural Killer (NK) cells, while reducing regulatory T cell infiltration to alleviate tumor-associated immunosuppression [6,7,8,9]. Blood-activating herbs like Salvia miltiorrhiza have been reported to inhibit angiogenesis by targeting the vascular endothelial growth factor (VEGF) pathway, thereby suppressing tumor growth [10]. Compounds such as matrine and paclitaxel can induce apoptosis or arrest the cell cycle in tumor cells, inhibiting tumor proliferation [11,12]. Clinically, TCM used as an adjunct to conventional therapies has shown potential to improve survival and reduce treatment-related adverse effects. These observations suggest that the antitumor effects of TCM are often closely associated with the regulation of immune responses, angiogenesis, stromal interactions, and other TME-related processes. Therefore, experimental platforms capable of reconstructing the dynamic and multicellular features of the TME are particularly important for mechanistic studies of TCM.

Despite these promising findings, the mechanisms underlying TCM remain insufficiently understood. The multi-component nature of herbal formulas, the dynamic interactions within the TME, and issues such as low bioavailability and complex pharmacokinetics contribute to a “black-box” phenomenon that limits mechanistic interpretation and standardization of TCM-based therapies [13]. These factors are key bottlenecks limiting the standardization and internationalization of TCM anti-cancer research.

Traditional animal models, while physiologically integrated, are constrained by species discrepancies, limited real-time observation, and ethical constraints [14]. Often constructed either based on empirical syndromes such as blood stasis and heat toxicity or single factors such as stress, making it difficult to replicate the complex and multidimensional nature of disease pathology. Consequently, these models are often inadequate for comprehensively evaluating the multi-component, multi-target, and microenvironment-dependent therapeutic effects of TCM, especially when dynamic interactions among tumor, stromal, vascular, and immune components are involved [15]. Two-dimensional (2D) cell cultures grossly oversimplify the tissue architecture and fail to recapitulate cell–cell and cell–matrix interactions, while patient-derived organoids often lack vascularization, immune components, and dynamic fluid environments essential for modeling the TME [16,17]. Microfluidic tumor microenvironment-on-a-chip platforms offer a promising alternative for addressing these challenges. By manipulating fluids within micron-scale channels, microfluidic systems enable the reconstruction of three-dimensional tumor models with controllable shear stress, nutrient gradients, and multicellular interactions. These platforms allow the co-culture of tumor cells, endothelial cells, immune cells, and stromal components in perfusable systems, enabling real-time observation of angiogenesis, immune-cell infiltration, and treatment responses under physiologically relevant conditions [18,19,20]. Such features are particularly valuable for TCM research, because they make it possible to analyze how complex herbal compounds or formulations act on multiple cellular compartments of the TME in a quantitative and controlled manner. However, the integration of microfluidic technology with TCM research remains at an early stage, and challenges persist in aligning the controlled analytical framework of microfluidics with the inherent complexity of TCM formulations.

In this review, we focus on how microfluidic TME-on-a-chip platforms can address several longstanding bottlenecks in TCM-based anticancer research, including insufficient mechanistic interpretation of multi-component therapies, the lack of physiologically relevant evaluation models, and the difficulty of capturing microenvironment-dependent drug responses. We first summarize recent advances in the construction of TME-on-a-chip systems and then discuss their emerging roles in evaluating the efficacy and mechanisms of TCM-derived compounds and formulations. Finally, we outline current technical limitations, standardization challenges, and future directions for integrating microfluidic technologies into mechanism-driven and precision-oriented TCM research.

## 2. Tumor Microenvironment

Tumor progression is increasingly recognized as a dynamic process governed not only by malignant cells themselves but also by the surrounding TME, which actively shapes disease progression, therapeutic response, and resistance. In line with the modern understanding of cancer as a disease characterized by multiple hallmark capabilities [21], the TME provides structural, biochemical, immunological, and metabolic support for tumor growth, invasion, metastasis, and therapeutic resistance [22,23]. Therefore, understanding the composition and functional interactions of the TME is essential for elucidating tumor biology and developing effective anticancer strategies. At the same time, because many antitumor effects of TCM are proposed to involve system-level regulation rather than direct cytotoxicity alone, the TME also provides a biologically relevant framework for interpreting TCM actions in oncology.

### 2.1. Key Components of the Tumor Microenvironment

**Figure 1:**
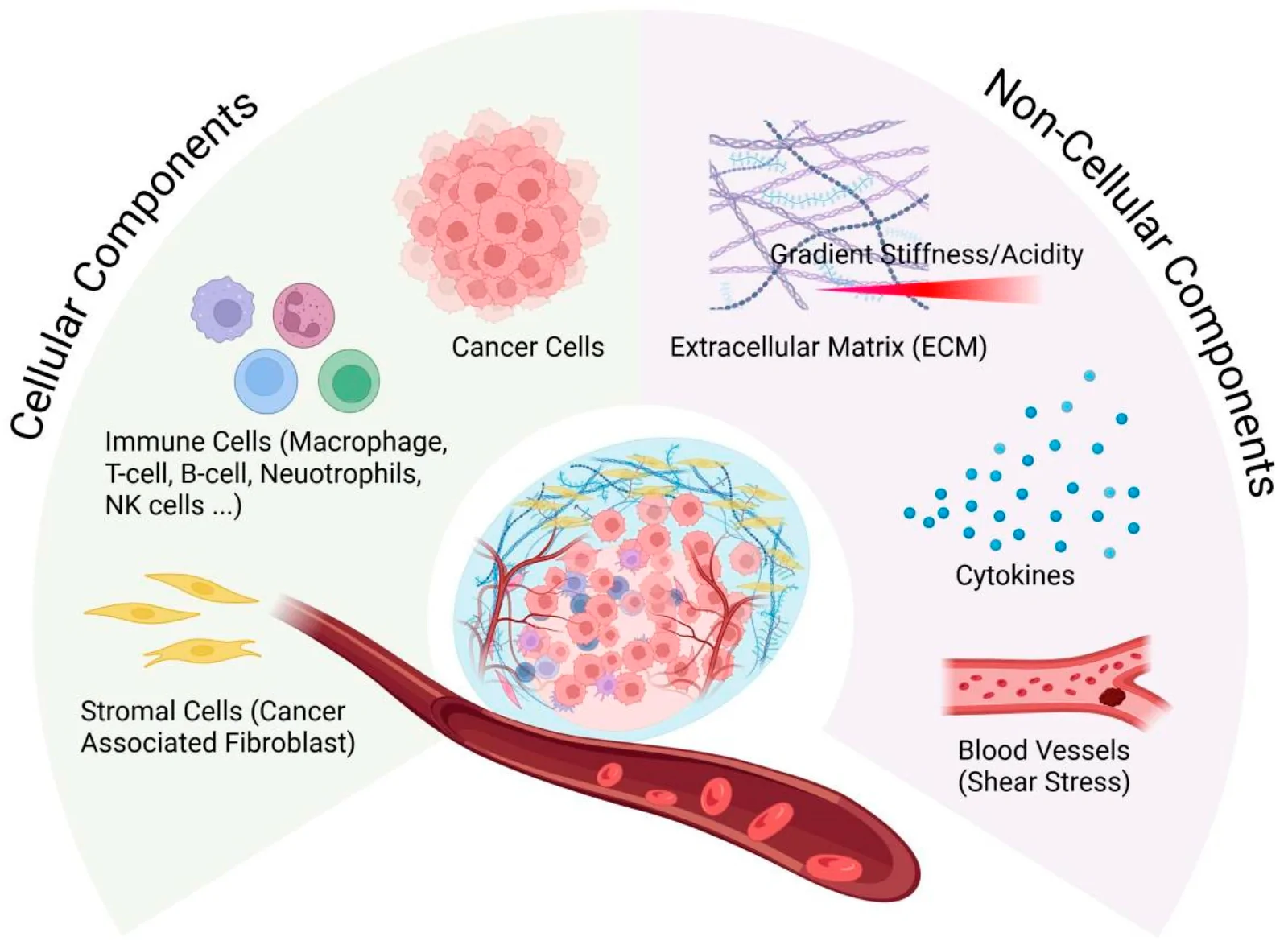
Composition of tumor microenvironment. (Created with BioRender.com).

The TME is a dynamic ecosystem composed of tumor cells, immune cells, stromal cells, vascular structures, extracellular matrix (ECM), soluble signaling factors, and metabolic products, all of which interact to regulate tumor progression and treatment response [24] (Figure 1).

At a functional level, the TME consists of interacting cellular and non-cellular elements. Tumor cells actively reprogram immune, stromal, and vascular compartments through cytokines, chemokines, growth factors, and extracellular vesicles, thereby promoting proliferation, invasion, angiogenesis, and immune escape [25,26]. Immune populations such as Tumor Associated Macrophages (TAMs) and Regulatory T cells (Tregs) often contribute to immunosuppression, whereas CAFs and endothelial cells support matrix remodeling, abnormal perfusion, barrier formation, and metastatic dissemination [27,28,29]. These cellular components function in a highly coordinated manner, and their reciprocal interactions are critical for establishing the pathological features of the TME. In parallel, non-cellular features such as abnormal vasculature, ECM remodeling, soluble signaling networks, hypoxia, acidosis, and metabolic gradients further shape tumor behavior and treatment response [30,31,32,33]. Together, these biochemical, mechanical, and metabolic interactions define the dynamic nature of the TME and create major challenges for conventional in vitro models.

### 2.2. The Tumor Microenvironment as a Therapeutic Target

Because the TME actively supports tumor survival, evolution, and treatment resistance, it has become an important therapeutic target in cancer treatment. Current TME-directed strategies mainly include immune modulation, anti-angiogenic therapy, stromal regulation, and interference with inflammatory or metabolic signaling. Representative examples include immune checkpoint inhibitors targeting the PD-1/PD-L1 (Programmed Cell Death Protein 1/Programmed Death-Ligand 1) and CTLA-4 (Cytotoxic T-Lymphocyte-Associated Protein 4) axes [34,35,36,37,38], anti-VEGF agents that suppress abnormal angiogenesis [39,40], and emerging therapies directed against TAMs, matrix remodeling, inflammatory cytokines, or immunosuppressive metabolism, such as CSF1R inhibitors [41,42], MMP inhibitors, IL-6 pathway antagonists, and IDO inhibitors [43,44,45]. These approaches reflect a shift from directly killing tumor cells alone to reshaping the supportive microenvironment, thereby highlighting the need for experimental models capable of capturing multicellular and context-dependent therapeutic effects (Table 1).

However, the therapeutic effects of TME-targeting strategies are strongly influenced by the heterogeneity, spatial organization, and dynamic evolution of the microenvironment. Conventional two-dimensional culture systems lack multicellular interactions, matrix architecture, fluid flow, and biochemical gradients, whereas animal models are limited by species differences, low throughput, and difficulty in real-time observation. These limitations are particularly relevant for TCM, whose antitumor actions often involve multi-component, multi-target, and system-level regulation of the TME. Therefore, more physiologically relevant experimental platforms are needed to reconstruct the complexity of the TME and to evaluate therapeutic responses in a controlled and dynamic manner. In this context, microfluidic tumor microenvironment-on-a-chip platforms provide a promising approach for reconstructing key TME features and bridging mechanistic research with pharmacological evaluation. These limitations are especially important when the research objective is not merely to measure tumor cell viability, but to investigate how TCM affects barrier transport, immune-cell behavior, stromal interactions, or therapy response within a dynamic microenvironment. Therefore, experimental systems that can reconstruct multicellular organization, perfusion, and biochemical gradients are particularly needed. Accordingly, in the following sections we distinguish between studies describing TME-regulating effects of TCM, studies developing microfluidic tumor models, and the more limited but particularly important studies that genuinely integrate both. These challenges are especially evident when the research aim is to investigate drug penetration, immune regulation, multicellular interactions, or synergistic therapeutic responses in the TME context.

## 3. The Role of Traditional Chinese Medicine in Regulating the Tumor Microenvironment

### 3.1. Traditional Chinese Medicine Perspective on Tumors

Traditional Chinese Medicine (TCM), with a history spanning thousands of years, has long interpreted disease through holistic frameworks such as Yin–Yang balance, the Five Elements, and the unity between the human body and its environment [46]. Classical TCM theory, as reflected in the *Huangdi Neijing*, emphasizes that disease arises from the disruption of internal balance and the weakening of the body’s resistance to pathogenic factors [47]. Although developed within a different epistemological system, this perspective conceptually parallels the modern view of cancer as a systemic and dynamic disease influenced by the tumor and its microenvironment.

In TCM, tumors are often associated with pathological patterns such as Qi stagnation, blood stasis, phlegm-dampness, and toxic heat [48]. Rather than focusing only on the local lesion, TCM emphasizes the interaction between the tumor and the host state. From a modern perspective, these concepts can be cautiously linked to processes such as abnormal perfusion, extracellular remodeling, chronic inflammation, immune suppression, and metabolic imbalance within the TME.

This systems-oriented therapeutic logic makes TCM particularly relevant to TME-directed cancer research. However, it also creates a methodological challenge, because many conventional experimental systems are poorly suited to evaluating multi-component and microenvironment-dependent effects. Therefore, the central methodological question is how to evaluate these multi-component and microenvironment-dependent effects in experimental systems that retain controllability while better recapitulating in vivo complexity. This challenge provides a direct rationale for the use of microfluidic tumor models.

### 3.2. Regulation of the Tumor Microenvironment by Chinese Herbal Medicine

Chinese herbal medicine (CHM) exerts antitumor effects, at least in part, through regulation of the tumor microenvironment, a process that can be understood through both traditional therapeutic principles and experimentally identifiable biological mechanisms. From the perspective of TCM theory, CHM may reinforce healthy Qi, resolve phlegm-dampness, clear heat and toxins, and promote circulation to remove stasis. In modern biomedical terms, these actions are increasingly associated with the regulation of key TME processes, including immune reprogramming, angiogenesis, stromal remodeling, metabolic adaptation, and inflammatory signaling. Accumulating evidence indicates that CHM displays multi-component, multi-target, and system-level characteristics in regulating the TME, rather than acting through a single molecular pathway [49,50,51,52,53,54]. In particular, questions related to penetration across stromal barriers, immune-cell recruitment, dynamic paracrine signaling, and treatment response under flow conditions remain difficult to resolve in conventional models.

#### 3.2.1. Immunomodulation

Immunomodulation is one of the most extensively studied mechanisms by which CHM remodels the TME. Qi-tonifying herbs such as *Astragalus membranaceus* and *Panax ginseng* are traditionally used to “reinforce healthy Qi.” Their active components, including Astragalus polysaccharides (APS), PG2, ginseng polysaccharides, and ginsenoside Rh2, have been shown to reverse immunosuppression by promoting M1 macrophage polarization, enhancing dendritic-cell maturation, improving antigen presentation, and stimulating T-cell responses [49,50,51,55,56,57,58,59]. For example, APS and related Astragalus polysaccharide fractions activate Toll-Like Receptor 4 (TLR4)-associated signaling and support both macrophage reprogramming and dendritic-cell function [55,57,58], whereas ginseng polysaccharides enhance CD8^+^ T-cell activity and relieve immunosuppressive metabolic constraints [56,59]. Ginsenoside Rh2 also promotes M1 polarization and suppresses invasion-related mediators such as VEGF, MMP-2, and MMP-9 [59]. Other compounds, such as celastrol, have also shown the ability to induce immunogenic cell death, downregulate PD-L1, and attenuate NF-κB-related inflammatory signaling, further supporting the immunoregulatory potential of CHM in the TME [60,61].

#### 3.2.2. Anti-Angiogenesis and Stromal Remodeling

CHM can also regulate the TME by suppressing abnormal angiogenesis and remodeling stromal barriers, which is conceptually consistent with the TCM principle of promoting circulation and resolving stasis. Baicalein, a major flavonoid from *Scutellaria baicalensis*, inhibits tumor angiogenesis through the TLR4/Hypoxia-Inducible Factor (HIF)-1β/VEGF signaling axis [62]. Aiphanol, isolated from *Smilax glabra*, exerts anti-angiogenic activity through dual inhibition of VEGFR2 and COX2 [63]. Beyond vascular regulation, stromal remodeling is another important target. Anisamide-modified red blood cell membrane-coated silybin-loaded nanoparticles (ARm@SNP) can specifically target CAFs, disrupt stromal and immunosuppressive barriers, and inhibit epithelial–mesenchymal transition, thereby facilitating drug penetration and reducing metastatic potential [64]. These findings indicate that CHM and CHM-derived formulations may enhance therapeutic efficacy not only by inhibiting vascular support but also by modifying the physical and cellular architecture of the TME.

#### 3.2.3. Metabolic Reprogramming

Metabolic reprogramming is another hallmark of the TME that can be modulated by CHM. Bruceine A has been shown to suppress the Warburg effect in pancreatic cancer through the PFKFB4/GSK3β signaling pathway, thereby limiting glycolytic metabolism and tumor growth [65]. In prostate cancer models, *Ganoderma tsugae* ethanol extract inhibits lipogenesis through regulation of the SREBP-1/androgen receptor axis, suggesting that CHM can also interfere with tumor-associated lipid metabolic reprogramming [66]. Because metabolic disturbances in tumors contribute to immune suppression, nutrient competition, and physicochemical stress within the TME, these observations further support the potential of CHM to reshape the tumor-supportive metabolic environment.

Overall, current evidence indicates that CHM regulates the TME through interconnected mechanisms involving immune modulation, angiogenesis inhibition, stromal remodeling, metabolic intervention, and inflammatory signaling [50,51,52,53,54,67]. However, these mechanisms are highly context-dependent and are often difficult to dissect using conventional static culture systems. This limitation is particularly important for TCM research, where multi-component exposure, spatial heterogeneity, and time-dependent responses may all influence therapeutic outcomes. Therefore, microfluidic tumor-on-a-chip platforms may provide an important technological bridge for translating TCM theory into measurable biological processes by enabling controllable, real-time, and physiologically relevant evaluation of CHM-mediated TME regulation (Table 2).

### 3.3. Synergistic and Efficacy-Enhancing Effects of Traditional Chinese Medicine in Cancer Therapy

In addition to direct TME regulation, TCM has also been reported to enhance the efficacy of conventional anticancer therapies, often through microenvironment-related mechanisms [68]. One important mechanism underlying these synergistic effects is the remodeling of the immunosuppressive TME and the enhancement of antitumor immunity. For example, *Astragalus membranaceus* (*Huangqi*) has been reported to enhance the antitumor efficacy of cisplatin in lung cancer models. Astragaloside IV improves cisplatin sensitivity by suppressing B7-H3 expression [69]. Compound *Sophora flavescens* Injection enhances the activity of sorafenib by reversing M2 macrophage-mediated immunosuppression and activating CD8^+^ T cells [70]. Similarly, the XIAOPI formula improves paclitaxel response by reducing CXCL1-mediated extracellular vesicle biogenesis and inhibiting PD-L1-associated M2 polarization [71].

Additional studies suggest that TCM may help overcome resistance through coordinated effects on EMT, metabolism, and drug transport. Representative examples include curcumin in combination with sorafenib, andrographolide with cetuximab, and Schisandrin B with 5-fluorouracil [72,73,74]. Ferroptosis-related sensitization has also been reported, as illustrated by β-elemene in combination with cetuximab and shikonin in cisplatin-resistant ovarian cancer models [75,76].

Collectively, these studies suggest that the efficacy-enhancing effects of TCM arise largely from its system-level regulation of the tumor microenvironment rather than from single-target activity alone. This feature is highly consistent with the emerging view that effective cancer therapy should not only eliminate malignant cells but also reprogram the surrounding ecological network that supports tumor survival, metastasis, immune escape, and drug resistance. However, these synergistic mechanisms still require validation in experimental models that can better capture the dynamic and multicellular nature of the TME.

## 4. Microfluidic Platforms for Investigating TCM–Tumor Microenvironment Interactions

Despite the growing evidence supporting TCM-mediated regulation of the TME and its synergy with conventional therapies, conventional experimental models remain insufficient to fully capture these dynamic and multicellular processes. This limitation underscores the need for advanced experimental platforms that can more faithfully reconstruct the spatial organization, dynamic interactions, and physicochemical heterogeneity of the TME. Among these emerging technologies, microfluidic chips have attracted increasing attention in cancer research. By enabling precise control over cellular composition, fluid flow, biochemical gradients, and tissue interfaces, microfluidic systems provide a promising bridge between conventional in vitro assays and in vivo models. In the context of TCM research, such platforms are particularly valuable because they offer a means to investigate how multi-component interventions modulate tumor cells and the surrounding microenvironment in a dynamic and integrated manner.

### 4.1. Microfluidic Technology and the Construction of Microfluidic Chips

Microfluidic technology enables the precise manipulation of fluids within microscale channels, where laminar flow and diffusion-dominated transport allow accurate control of cellular organization, biochemical gradients, and mechanical stimuli [77]. Through microfabrication techniques, diverse cell types, extracellular matrices, and perfusion systems can be integrated into a single platform, thereby reconstructing key structural and functional features of the TME under highly controllable conditions. Using microfabrication techniques such as photolithography and thin-film deposition, complex networks of microchannels, chambers, and reaction units can be integrated onto chip substrates. By applying pressure-driven flow or electroosmotic forces, minute volumes of fluid can be transported through these networks, enabling the integration of sample preparation, mixing, reaction, separation, and detection on a single platform [78]. In addition, the short diffusion distances and efficient heat transfer at this scale facilitate rapid and reliable biochemical reactions. Owing to these features, microfluidic systems have become increasingly important in biomedical research and drug development, particularly for modeling complex biological systems in which dynamic transport, multicellular interactions, and microenvironmental regulation must be considered simultaneously.

### 4.2. Advantages of Microfluidic Chips

Recent advances in therapies targeting the TME, including immune checkpoint inhibitors and anti-angiogenic agents, have substantially changed the landscape of cancer treatment. However, tumor heterogeneity and adaptive resistance continue to contribute to the high attrition rate of anticancer drug development [28]. One important reason for the discrepancy between promising preclinical results and limited clinical success lies in the limitations of conventional experimental models. Subcutaneous xenograft models, which have long been widely used in anticancer research, fail to faithfully recapitulate the endogenous TME [79]. When human tumor cells are implanted subcutaneously into immunodeficient mice, the resulting stromal composition, immune contexture, and vascular architecture differ markedly from those of native human tumors. Such differences can affect tumor behavior even at the molecular level, including the expression of vascular and microenvironment-related proteins [80].

Traditional animal models also provide limited temporal and spatial resolution. In many cases, it remains difficult to visualize dynamic interactions between cancer cells and stromal components in real time or to isolate the contribution of individual microenvironmental factors to tumor progression [81]. Although patient-derived organoid models offer strong potential for personalized prediction, their establishment is often labor-intensive, time-consuming, and associated with variable success rates [82]. Therefore, there is a clear need for experimental platforms that can reproduce key features of in vivo tumor development with both high fidelity and operational efficiency.

Since the beginning of the 21st century, microfluidic chips have emerged as a promising solution to this gap by providing highly controllable, physiologically relevant, and ethically advantageous in vitro models [83]. Through precise regulation of fluid flow, these systems can continuously supply nutrients and oxygen while removing metabolic waste, thereby mimicking aspects of blood circulation and supporting long-term cell culture [84]. Moreover, microfluidic platforms can be designed to model not only single-organ pathophysiology but also complex inter-organ communication, which improves the throughput and reliability of drug screening and supports the development of precision medicine strategies (Table 3). For studies focused on TCM, these advantages are especially relevant because they allow researchers to examine the transport, distribution, metabolism, and coordinated biological effects of multi-component interventions under conditions that more closely resemble the native tumor milieu.

### 4.3. Microfluidic Platforms for Modeling Key TME Features Relevant to TCM Research

A central challenge in TCM-oriented cancer research is to understand how multiple bioactive components are transported, metabolized, and spatially distributed within the heterogeneous TME before reaching their cellular targets, and how these processes influence tumor-stromal-immune interactions, therapeutic efficacy, and safety. From this perspective, microfluidic platforms are not merely engineering tools, but biologically informative systems that can be tailored to address specific scientific questions in TCM-related TME research. Accordingly, the following sections are organized around key aspects of the TME that are particularly relevant to TCM studies, including multicellular interactions, physicochemical microenvironmental cues, and organ-level or inter-organ modeling, while highlighting the advantages of microfluidic systems over conventional models.

#### 4.3.1. Modeling Multicellular Interactions Within the TME on Chips

A defining feature of the TME is its multicellular organization, in which tumor cells continuously interact with CAFs, endothelial cells, and diverse immune populations rather than functioning in isolation [88]. These dynamic cellular interactions critically shape tumor growth, angiogenesis, immune evasion, and therapeutic response. Through microfabrication techniques, diverse cell types, extracellular matrices, and perfusion systems can be integrated into a single platform, thereby reconstructing key structural and functional features of the TME under highly controllable conditions

**Figure 2:**
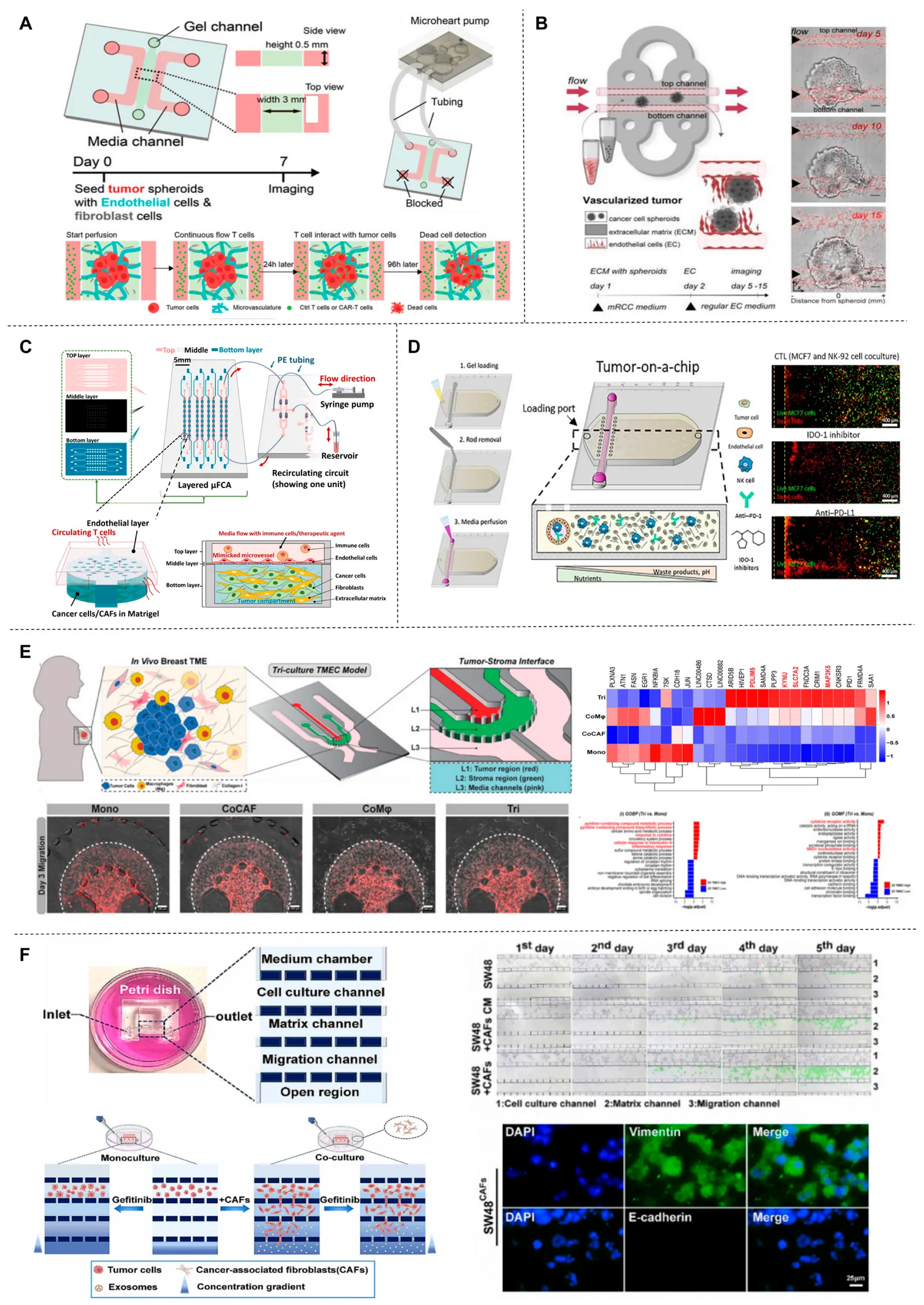
Representative microfluidic platforms for multicellular tumor microenvironment reconstruction. (**A**) A vascularized tumor spheroid microfluidic chip containing tumor spheroids, fibroblasts, and vascular cells, used to evaluate the effects of fibroblast distribution on tumor vascularization, vessel perfusability, and CAR–T–cell recruitment, cytotoxicity, and inflammatory cytokine secretion under continuous-flow conditions. Reproduced with permission from Ref. [89]. Copyright 2022, Wiley–VCH GmbH. (**B**) A microfluidic co–culture platform incorporating metastatic renal cell carcinoma spheroids and endothelial cells in a collagen matrix, used to investigate tumor angiogenesis, vessel functionality, drug delivery, and the response to vascular-targeted therapy. Reproduced with permission from Ref. [90]. Copyright 2025, Ivyspring International. (**C**) A recirculating circuit-integrated PDMS-based microfluidic cell array (μFCA) enabling continuous immune–cell recirculation for the analysis of interactions among reconstructed tumor microenvironment components, immune cells, and immunotherapeutic interventions. Reproduced with permission from Ref. [91]. Copyright 2024, The Royal Society of Chemistry. (**D**) A tumor–on–a–chip model comprising collagen-embedded tumor cells and NK cells adjacent to a perfused endothelialized lumen, used to assess the role of tumor-associated stress in NK–cell exhaustion and the effects of immunomodulatory agents on functional restoration. Reproduced with permission from Ref. [92]. Copyright 2021, American Association for the Advancement of Science. (**E**) A PDMS–based tumor microenvironment-on-a-chip model with spatially organized tumor and stromal regions, used to monitor stromal–immune–tumor crosstalk, identify pathways associated with tumor progression, and evaluate the inhibitory effects of small-molecule compounds on tumor invasion. Reproduced with permission from Ref. [93]. Copyright 2025, Wiley–VCH GmbH. (**F**) A microfluidic co–culture system for colorectal cancer cells and patient–derived CAFs, used to analyze CAF–mediated regulation of cancer-cell proliferation, invasion, epithelial–mesenchymal transition, and gefitinib sensitivity, including the contribution of CAF–derived exosomes. Reproduced with permission from Ref. [94]. Copyright 2024, Springer-Verlag GmbH Austria, part of Springer Nature.

Microfluidic vascularized tumor models have been used to investigate tumor–vascular interactions. Wan et al. established a vascularized tumor spheroid chip in which tumor spheroids and vascular cells were cultured within a perfusable three-dimensional microenvironment. The platform generated functional microvascular networks and enabled real-time analysis of Chimeric Antigen Receptor T (CAR-T)-cell recruitment, tumor infiltration, cytokine secretion, and drug penetration [89] (Figure 2A). Similarly, Skubal et al. used a co-culture chip containing metastatic renal carcinoma spheroids and endothelial cells embedded in collagen matrix, demonstrating that Prostate-Specific Membrane Antigen (PSMA) promoted angiogenic sprouting whereas bevacizumab effectively disrupted vascular network formation [90] (Figure 2B).

These vascularized and perfusable systems are particularly relevant to TCM research, because many TCM interventions are proposed to influence circulation, tissue penetration, angiogenesis, and microenvironmental homeostasis. Microfluidic chips therefore provide a useful framework for determining whether specific herbal extracts or compound formulations alter vascular permeability, improve intratumoral delivery, or enhance the spatial accumulation of active constituents within three-dimensional tumor tissues.

Microfluidic platforms have also been widely used to reconstruct immune-related components of the TME. Chi et al. developed a microfluidic array chip that supported continuous immune-cell recirculation while integrating tumor cells, endothelial cells, and fibroblasts into a structured TME model. Using circulating CD8^+^ T cells, they demonstrated that tumor-associated endothelium restricted T-cell infiltration, whereas anti-PD-L1 treatment partially restored this process; the presence of CAFs further suppressed T-cell infiltration and tumor cell apoptosis [91] (Figure 2C). Likewise, Ayuso et al. established a breast tumor-on-a-chip incorporating NK cells and endothelialized microvessels. The platform generated physiologically relevant nutrient gradients and demonstrated that metabolic stress induced persistent NK-cell exhaustion characterized by increased PD-1, IDO-1, and CTLA-4 expression [92] (Figure 2D).

Compared with conventional 2D assays, these systems permit real-time observation of immune-cell infiltration, migration, cytotoxic activity, and functional exhaustion under dynamic flow conditions. This capability is especially valuable for evaluating TCM strategies aimed at restoring host immune competence, because it allows investigators to assess not only changes in tumor burden, but also whether a given intervention alleviates immune exhaustion, improves stromal penetration, and enhances antitumor immune activity in a more physiologically relevant context.

Beyond vascular and immune compartments, microfluidic chips have also been developed to reproduce stromal interactions within the TME. Kalpana Ravi et al. developed a multicomponent tumor chip containing CAFs and macrophages embedded in collagen/Matrigel matrix. Combined with single–cell RNA sequencing, the model demonstrated that CAF–macrophage crosstalk promoted tumor invasion and immune evasion through activation of the kynurenine pathway [93] (Figure 2E). Similarly, Li et al. further established a pump-free colorectal cancer-CAF co-culture chip and showed that CAF-derived exosomes enhanced proliferation, epithelial–mesenchymal transition (EMT), invasion, and resistance to gefitinib, with these effects varying according to CAF [94] (Figure 2F).

Taken together, by reconstructing vascular, immune, and stromal interactions with improved physiological relevance, microfluidic platforms provide an important technological basis for future mechanistic studies exploring how multi-component TCM interventions regulate the TME. As more studies directly combine herbal medicines with these platforms, microfluidic technology is expected to become an increasingly valuable tool for validating the systems-level mechanisms proposed for TCM in cancer therapy.

#### 4.3.2. Tumor Microenvironment Chips with Diverse Physicochemical Characteristics

The TME is defined not only by its cellular composition but also by a distinct physicochemical landscape characterized by hypoxia, nutrient deprivation, altered ECM mechanics, and abnormal fluid flow [95]. These environmental cues are also highly relevant to TCM research because many herbal compounds are proposed to regulate hypoxia-associated signaling, stromal remodeling, tumor perfusion, and metabolic reprogramming rather than acting exclusively through direct cytotoxicity [96]. Microfluidic platforms provide unique opportunities to independently control these physicochemical parameters, thereby enabling mechanistic evaluation of how TCM interventions function under defined TME conditions.

**Figure 3:**
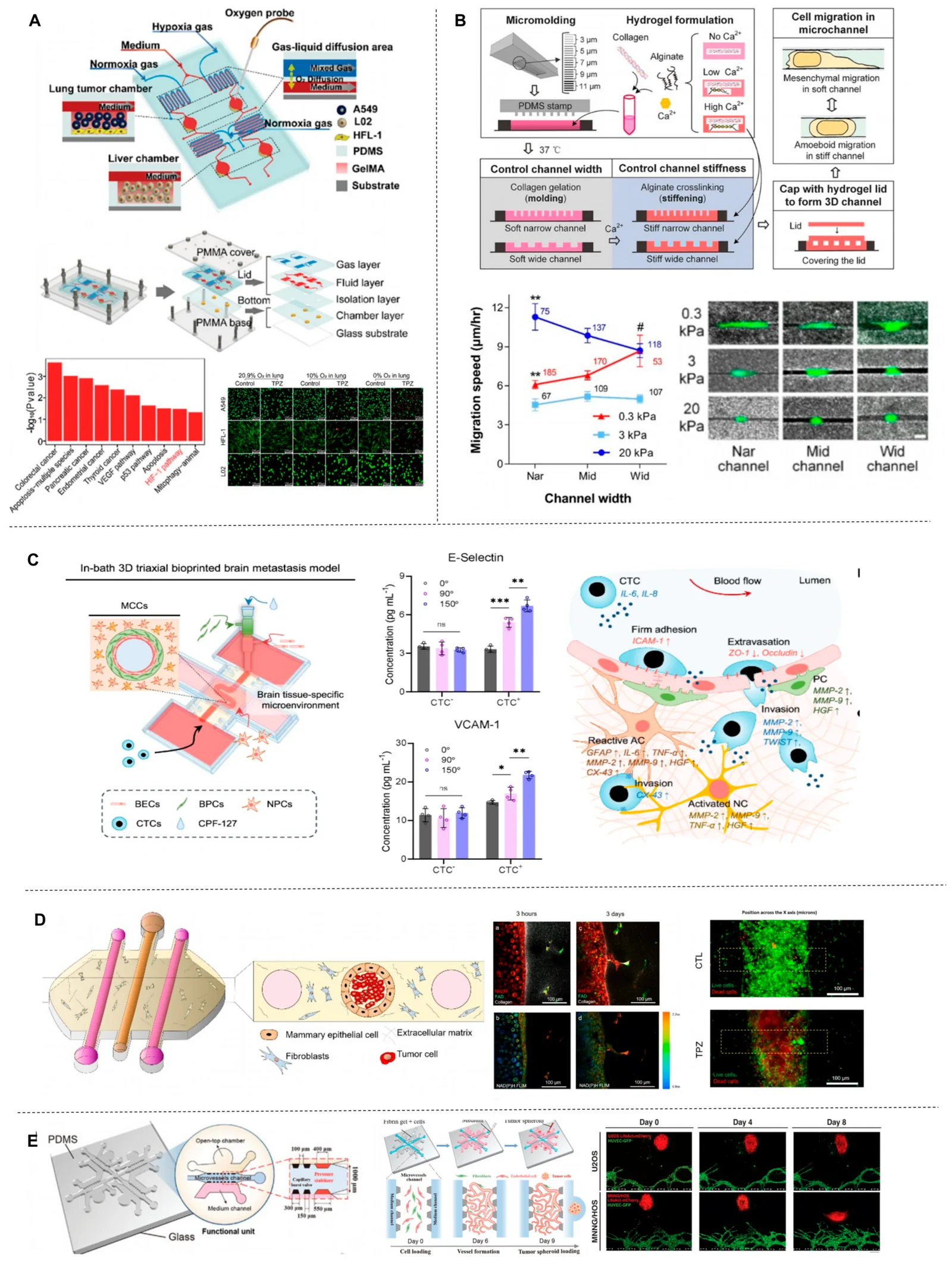
Representative microfluidic platforms for simulating physicochemical characteristics of the tumor microenvironment. (**A**) A three-dimensional co-culture multiorgan microfluidic (3D-CMOM) platform with controllable oxygen levels, used to investigate hypoxia-driven lung cancer–liver metastasis and to evaluate antitumor efficacy and off-target toxicity in a multiorgan setting. Reproduced with permission from Ref. [97]. Copyright 2021, American Chemical Society. (**B**) A collagen–alginate hydrogel microchannel chip with tunable stiffness, used to characterize matrix mechanics and to evaluate stiffness-regulated mesenchymal-to-amoeboid transition in MDA-MB-231 breast cancer cells. (Two-way ANOVA; mean ± SEM; ** *p* < 0.01, relative to control groups which were labeled as #.) Reproduced with permission from Ref. [98]. Copyright 2019, American Chemical Society. (**C**) A three-dimensional bioprinted multilayered cerebrovascular conduit model with variable vascular curvature, used for fluid-dynamic analysis and for investigating the effects of curvature-dependent shear stress on tumor-cell adhesion, endothelial disruption, and extravasation. Reproduced with permission from Ref. [99]. Copyright 2023, Springer Nature. (* *p* < 0.05, ** *p* < 0.01, *** *p* < 0.001). (**D**) A breast cancer organoid-on-a-chip platform designed to generate nutrient and oxygen gradients for modeling metabolic heterogeneity, hypoxia adaptation, and region-specific drug responses in ductal carcinoma in situ (DCIS). Reproduced with permission from Ref. [100]. Copyright 2018, Elsevier. (**E**) A human tumor–perivascular (hTPV) chip used to reconstruct tumor-specific hierarchical microvascular networks and physiological flow conditions for analyzing angiogenesis, tumor-cell migration, metastatic potential, and responses to anti-angiogenic therapy. Reproduced with permission from Ref. [101]. Copyright 2024, Wiley-VCH GmbH.

Hypoxia is one of the most prominent physicochemical features of solid tumors and a well-established driver of malignant progression. Arising from rapid tumor growth and insufficient oxygen supply, hypoxia promotes EMT, invasive behavior, and treatment resistance. Zheng et al. developed a three-dimensional co-culture multiorgan microfluidic (3D-CMOM) chip with controllable oxygen levels to model lung tumor-liver interactions. Using this platform, they demonstrated that hypoxia promoted metastatic behavior through HIF-1α-mediated activation of EMT-associated transcription factors, including Snail1 and Snail2, and that this effect was further enhanced by fibroblasts. The system was also applied to drug screening, revealing that SYP-5 exhibited antitumor efficacy with relatively low toxicity, whereas IDF-11774 produced notable hepatotoxicity [97] (Figure 3A). Such hypoxia-controlled models may therefore provide an appropriate platform for determining whether TCM formulations suppress hypoxia-driven metastasis or enhance therapeutic efficacy by modulating hypoxia-responsive pathways.

In addition to oxygen availability, mechanical cues such as ECM stiffness and fluid shear stress exert profound effects on tumor-cell phenotype and dissemination. Wang et al. designed a collagen–alginate microchannel chip with tunable stiffness ranging from 0.3 to 20 kPa and controllable geometric features to mimic ECM mechanics. In MDA-MB-231 breast cancer cells, increased matrix stiffness alone induced a transition toward amoeboid migration, whereas softer matrices maintained a mesenchymal phenotype [98] (Figure 3B). This type of platform is particularly useful for studying how physicochemical properties shape invasion strategies, and may also be valuable for evaluating whether TCM-derived compounds or formulations modulate matrix remodeling, stromal contractility, or migration mode within the TME.

Abnormal hemodynamic conditions represent another important aspect of the physicochemical TME. Park et al. showed that vascular curvature altered local flow patterns and shear stress, thereby increasing tumor-cell adhesion through upregulation of endothelial adhesion molecules such as E-selectin and VCAM-1, disrupting endothelial integrity, and promoting metastatic extravasation [99] (Figure 3C). These findings highlight the importance of microvascular geometry in regulating tumor dissemination and further support the use of microfluidic devices for mechanistic studies of tumor–vascular interactions under physiologically relevant flow conditions.

Microfluidic chips are also well suited to reproduce metabolic gradients within tumors. Ayuso et al. developed a breast cancer organoid chip capable of generating oxygen and nutrient gradients that mimic those found in poorly perfused tumor regions. Under hypoxic and nutrient-deprived conditions, ductal carcinoma in situ (DCIS) cells displayed enhanced glycolytic activity and increased HIF-1α signaling. In the same model, the hypoxia-activated prodrug tirapazamine selectively eliminated cells located in low-oxygen regions, supporting the utility of this platform for testing therapies targeting metabolically heterogeneous tumor niches [100] (Figure 3D). Such systems provide an opportunity to investigate whether therapeutic interventions, including multi-component TCM formulations, can alter metabolic adaptation across distinct microenvironmental zones rather than acting solely through direct cytotoxicity.

More recently, Du et al. reported a human tumor–perivascular (hTPV) chip that generated a hierarchical microvascular network within a vascularized tumor organoid model. Using this system, the authors found that highly metastatic tumor cells promoted angiogenesis and preferentially migrated toward the vasculature through activation of Notch signaling. Although anti-angiogenic treatment reduced vessel formation, inhibition of Notch signaling was additionally required to suppress tumor dissemination, underscoring the value of combination strategies for controlling both angiogenesis and metastatic spread [101] (Figure 3E). This type of vascularized chip may also offer a relevant platform for evaluating interventions aimed at regulating tumor perfusion, vascular remodeling, and metastatic behavior in an integrated manner.

Taken together, microfluidic chips that incorporate physicochemical features of the TME, including oxygen gradients, nutrient stress, ECM mechanics, and hemodynamic forces, substantially improve the physiological relevance of in vitro tumor models. By combining three-dimensional structure, controlled perfusion, and multicellular interactions, these systems enable real-time analysis of tumor adaptation to hostile microenvironmental conditions and provide a practical platform for mechanistic studies and drug evaluation, including the investigation of complex multi-component therapies.

#### 4.3.3. Organoid/Organ-on-a-Chip Systems

Organoids are three-dimensional tissue models derived from stem cells or primary cells that self-organize under defined in vitro culture conditions into structures partially recapitulating the cellular composition, spatial architecture, and selected functions of native organs [102]. Organ-on-a-chip (OOC) systems further integrate living human cells with engineered microenvironments, structural boundaries, and dynamic perfusion to mimic organ-level physiological or pathological states [103]. Compared with conventional two-dimensional cultures and many animal models, these platforms provide improved human relevance and allow more precise interrogation of intercellular communication, tissue function, and drug responses.

For TCM-related research, organoid and OOC technologies are particularly noteworthy because they offer experimentally tractable models for studying organ-specific functions as well as inter-organ communication. Many classical TCM theories emphasize the functional coordination among visceral systems, and these concepts, although historically described in a qualitative framework, are increasingly approachable through bioengineered human tissue models. Rather than serving as direct equivalents of traditional concepts, organoids and OOC systems can be regarded as modern experimental platforms for examining the physiological and pathological bases underlying systemic regulation, metabolism, transport, and organ crosstalk.

**Figure 4:**
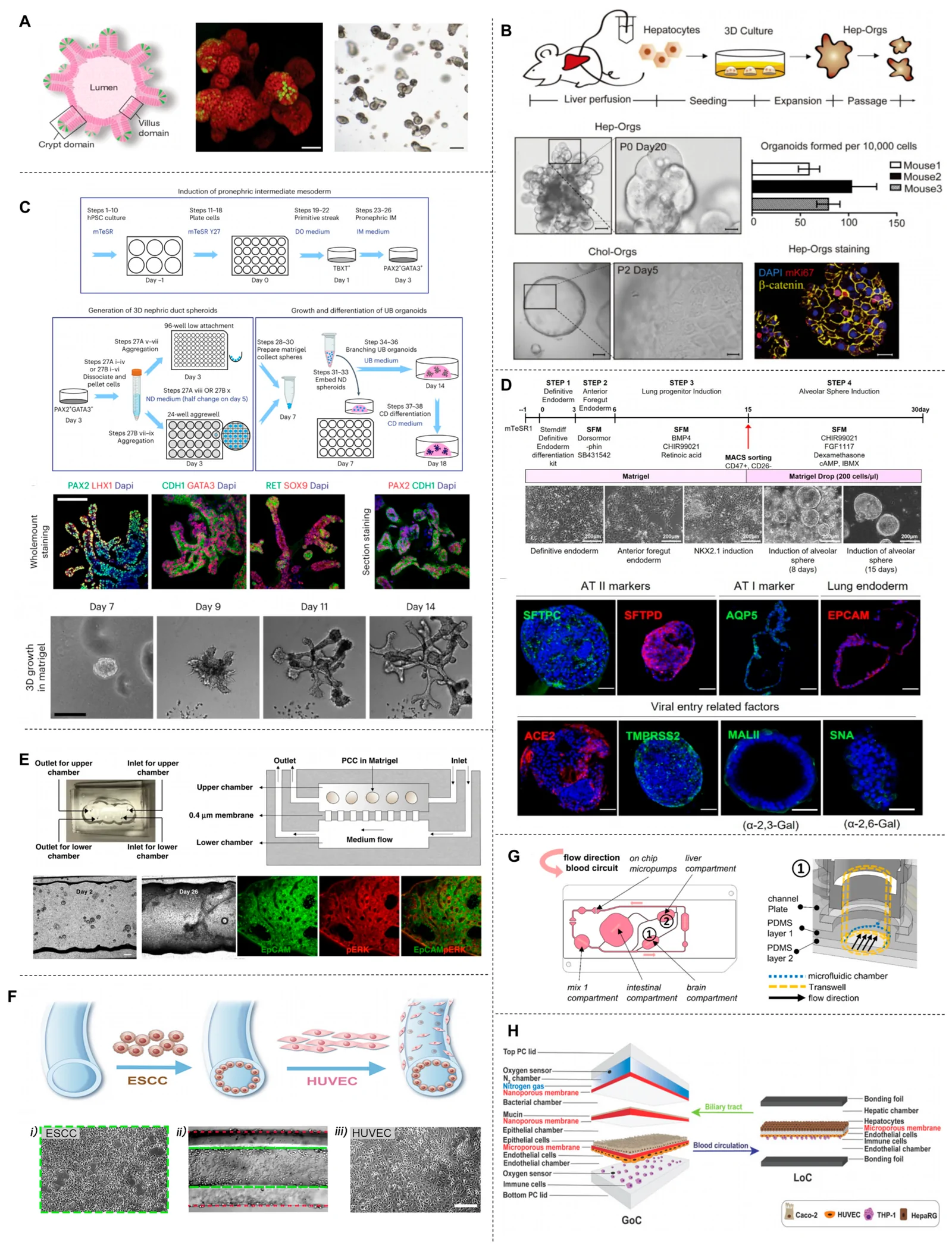
Representative organoid and organ-on-a-chip platforms for modeling organ-specific and cross-organ physiology. (**A**) Generation of intestinal crypt–villus organoids from Lgr5^+^ intestinal stem cells, used to model intestinal epithelial renewal, barrier function, and nutrient absorption. Reproduced with permission from Ref. [105]. Copyright 2009, Springer Nature. (**B**) Establishment of liver organoids from human induced pluripotent stem cells (hiPSCs) and primary hepatocytes for modeling hepatic differentiation, metabolism, and liver-specific functions. Reproduced with permission from Ref. [106]. Copyright 2019, BMJ. (**C**) Directed differentiation of ureteric bud and collecting duct organoids from human pluripotent stem cells (hPSCs), used to study renal development and water-transport-related epithelial structures. Reproduced with permission from Ref. [108]. Copyright 2023, Springer Nature. (**D**) Directed differentiation of alveolar organoids from pluripotent stem cells, generating alveolar type I and type II epithelial cells for modeling lung development and alveolar function. Reproduced with permission from Ref. [111]. Copyright 2023, Taylor & Francis Group. (**E**) A patient-derived pancreatic cancer-on-a-chip platform recapitulating key tumor microenvironment features, used to evaluate stromal targeting and chemotherapy response. Reproduced with permission from Ref. [112]. Copyright 2022, Springer Nature. (**F**) A hydrogel hollow-microfiber-based esophageal squamous cell carcinoma (ESCC) chip used for functional modeling of tumor invasion, chemoresistance, and microenvironment-associated pathogenic regulation. Reproduced with permission from Ref. [113]. Copyright 2024 Elsevier. Bright-field microscopic images showing two cell lines adhering to the hydrogel layer. The plane graph of (**i**) ESCC cells and (**iii**) HUVECs settled in (**ii**) the inner and outer walls of fibers, respectively. Scale bar, 200 μm. (**G**) Construction of the HUMIMIC Chip4 brain–liver organ-on-a-chip platform from HUMIMIC101 hiPSCs, enabling integrated modeling of blood–brain barrier function and liver metabolism under dynamic perfusion. Reproduced with permission from Ref. [114] Copyright 2022, MDPI. (**H**) A coupled HuMiX gut-on-a-chip and Dynamic42 liver-on-a-chip dual-organ platform used to reconstruct the gut–liver axis and to analyze microbiota-associated drug metabolism and inter-organ transport. Reproduced with permission from Ref. [115]. Copyright 2024, Wiley-VCH GmbH.

A range of organoid systems has already been established to model major organs relevant to drug absorption, metabolism, excretion, and tumor progression. Intestinal organoids derived from Lgr5^+^ intestinal stem cells or human induced pluripotent stem cells (hiPSCs) can self-organize into crypt–villus-like structures in extracellular matrix-supported cultures supplemented with factors such as R-spondin 1, epidermal growth factor (EGF), and Noggin, thereby reproducing key features of intestinal epithelial renewal, nutrient absorption, and barrier function [104,105] (Figure 4A). Similarly, liver organoids can be generated from hiPSCs or primary hepatocytes. Through sequential induction with developmental cues including Activin A, Wnt/β-catenin agonists, hepatocyte growth factor (HGF), and fibroblast growth factors (FGFs), hiPSCs can be directed toward hepatocyte-like cells, whereas primary hepatocytes can form self-renewing liver organoids under defined growth-factor conditions [106] (Figure 4B). These models provide useful tools for studying drug metabolism, detoxification, and liver-specific injury.

Kidney and lung organoids further extend this framework to additional organ functions closely associated with systemic homeostasis. Kidney organoids mimicking ureteric bud and collecting duct development can be derived from human pluripotent stem cells (hPSCs) through stepwise induction with CHIR99021, FGFs, retinoic acid, and related morphogens, ultimately generating branched AQP2^+^ structures relevant to water transport and renal development [107,108] (Figure 4C). In parallel, alveolar organoids can be generated from hiPSCs or embryonic stem cells by directing differentiation through definitive endoderm and anterior foregut lineages toward lung progenitors, followed by three-dimensional culture in Matrigel with factors such as CHIR99021, FGF7, and dexamethasone-containing supplements. These organoids contain alveolar type I and type II epithelial cell populations and are therefore suitable for modeling gas-exchange-related lung biology and pulmonary injury [109,110,111] (Figure 4D). Collectively, these organoid systems provide human-relevant platforms for establishing physiological baselines in organ function and for evaluating how disease or therapeutic intervention alters tissue-specific behavior.

Despite these advantages, single-organoid systems remain limited in their ability to reproduce the structural complexity and dynamic microenvironment of tumors. OOC platforms address this limitation by incorporating stromal components, tissue interfaces, and fluid flow. Haque et al. developed a pancreatic cancer-on-a-chip model integrating patient-derived organoids with a profibrotic stromal compartment and immune cells. This platform reproduced essential aspects of the pancreatic tumor microenvironment and demonstrated that stromal targeting enhanced the efficacy of chemotherapy [112] (Figure 4E). Likewise, Shi et al. constructed a hydrogel-based esophageal squamous cell carcinoma (ESCC) chip with tubular architecture to model the epithelial–vascular interface, enabling functional analysis of tumor invasion, chemoresistance, and microenvironment-associated pathogenic factors [113] (Figure 4F). These studies illustrate how OOC systems can bridge the gap between organoid biology and tumor microenvironment modeling by restoring spatial structure and dynamic tissue interactions.

Beyond single-organ disease models, multi-organ chips are of particular importance for studying systemic pharmacology and cross-organ regulation. Advanced platforms such as the HUMIMIC brain–liver chip integrate hiPSC-derived brain microvascular endothelial-like cells with multicellular liver spheroids composed of hepatocyte-like cells, endothelial cells, and mesenchymal stromal cells under dynamic perfusion, thereby linking a blood–brain barrier module with a metabolic liver module in a common circulation system [114] (Figure 4G). Similarly, Lucchetti et al. established a coupled gut–liver axis model by connecting the HuMiX gut-on-a-chip with the Dynamic42 liver-on-a-chip. Using intestinal epithelial, immune, endothelial, and hepatic cell types together with *Escherichia coli*, this platform reproduced key features of the gut–liver interface and enabled simulation of irinotecan metabolism under pathological conditions [115] (Figure 4H). Such systems are especially valuable for investigating absorption, first-pass metabolism, microbiota-related transformation, systemic transport, and organ-specific toxicity in an integrated manner.

From the perspective of TCM research, organoid and OOC technologies may provide a more suitable framework than traditional reductionist assays for evaluating complex interventions characterized by multi-component composition, multi-target activity, and systemic regulation. In particular, multi-organ platforms offer an opportunity to investigate how herbal formulas or bioactive compounds are transformed, redistributed, and functionally coupled across different organ systems. Although these models remain simplified representations of human physiology, they constitute an important bridge between traditional holistic concepts and modern mechanistic research by enabling quantitative, human-relevant evaluation of organ-specific and cross-organ therapeutic effects.

## 5. Key Scientific Questions Addressed by Microfluidic Platforms in TCM Anti-Tumor Research

Although the specific applications of microfluidic technology in TCM are discussed in the following subsections, its significance in this field extends well beyond miniaturization, automation, or reduced reagent consumption. In contrast to conventional static assays, microfluidic platforms allow dynamic control of concentration gradients, spatiotemporal exposure, interfacial transport, cell–cell and tissue–tissue interactions, and continuous perfusion. These features make microfluidics particularly valuable for TCM research, where therapeutic effects often arise from multicomponent composition, multitarget regulation, context-dependent activity, and metabolism-associated transformation. Therefore, the main contribution of microfluidic systems to TCM research is not simply improved experimental efficiency, but the ability to generate mechanistic insights that are difficult to obtain using traditional multiwell-plate or endpoint-based methods.

At the same time, current microfluidic studies in TCM remain heavily biased toward single natural compounds or simplified extracts, whereas investigations of classical formulae and multi-herb compatibility are still scarce. This is an important limitation because the core theory of TCM emphasizes formula compatibility, synergistic interactions, and toxicity attenuation through herb pairing and combinatorial use. Accordingly, the discussion below not only summarizes representative applications, but also critically evaluates what new mechanistic understanding these platforms have enabled and where important gaps remain.

### 5.1. Microfluidic Screening of TCM-Derived Anti-Cancer Agents and Combinatorial Effects

**Figure 5:**
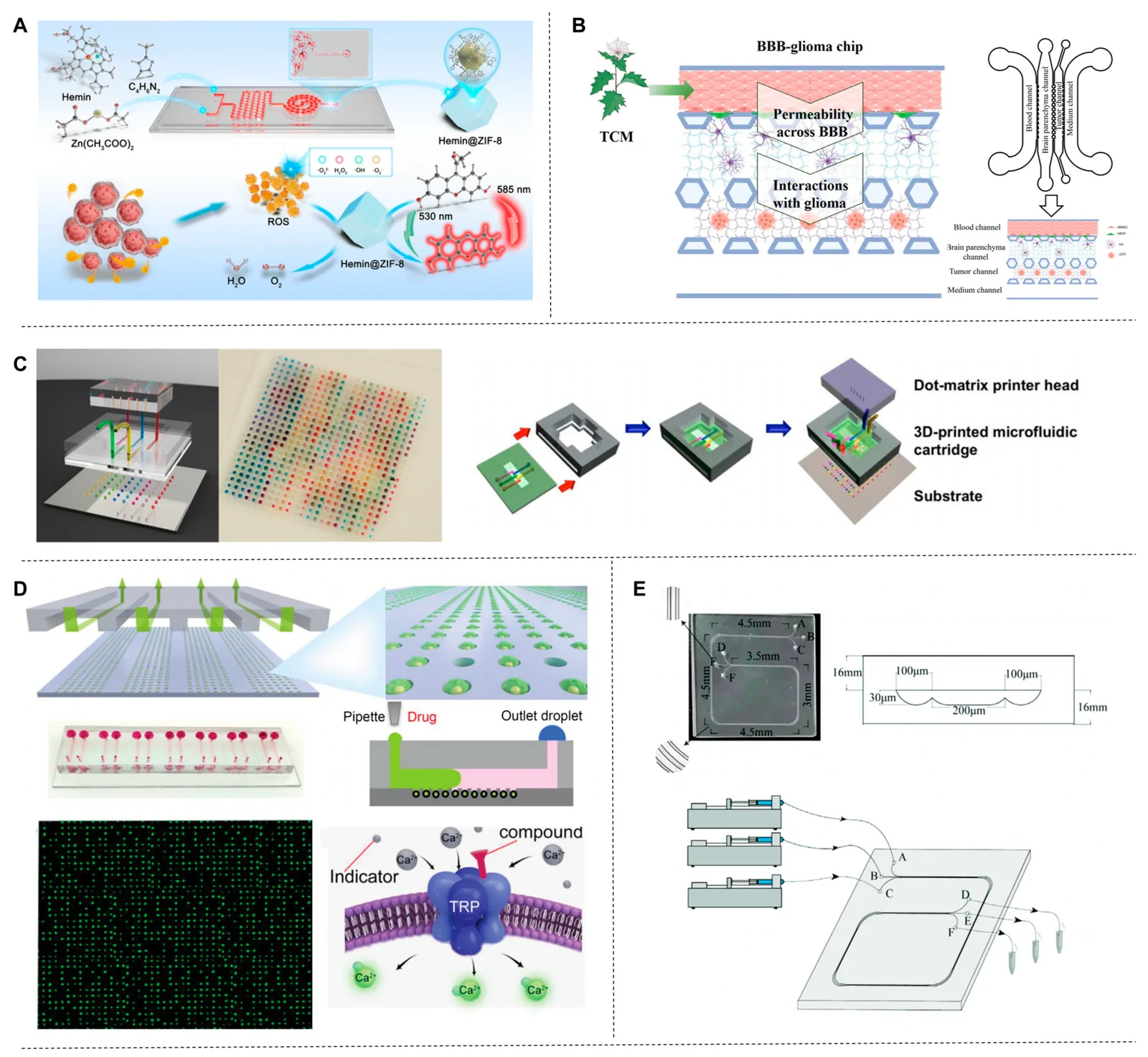
Representative microfluidic platforms for TCM-related anticancer drug screening. (**A**) A microfluidic platform for the synthesis of hemin@ZIF-8 nanozymes and their application in intracellular ROS detection, enabling dynamic functional evaluation in anticancer drug screening. Reproduced with permission from Ref. [116]. Copyright 2024, The Royal Society of Chemistry. (**B**) A blood–brain barrier (BBB)-U251 microfluidic chip used to evaluate the anti-glioma activity of TCM-derived components in a physiologically relevant in vitro model. Reproduced with permission from Ref. [118]. Copyright 2022, Elsevier B.V. (**C**) A microfluidic print-to-screen (P2S) platform for high-throughput combinatorial chemotherapy screening, enabling parallel drug testing with reduced reagent consumption. Reproduced with permission from Ref. [120]. Copyright 2015, American Chemical Society. (**D**) A single-cell microfluidic assay used to screen transient receptor potential (TRP) channel modulators from Murraya species while reducing false-positive rates associated with bulk assays. Reproduced with permission from Ref. [121]. Copyright 2020, Wiley-VCH Verlag GmbH & Co. KGaA. (**E**) A microfluidic laminar-flow chip integrated with dual-target competitive screening for the identification of G-quadruplex-targeting antitumor compounds from Macleaya cordata extracts. Reproduced with permission from Ref. [122]. Copyright 2021, The Royal Society of Chemistry.

A key question in TCM-based anti-cancer research is how to efficiently identify active compounds, combinations, or formulations with meaningful efficacy while capturing the dynamic and multicomponent nature of their pharmacological effects. Conventional screening approaches, however, often rely on endpoint-based measurements and simplified culture conditions, which limit their ability to capture dynamic cellular responses and the complex pharmacological behaviors of natural products. Microfluidic technology offers an alternative strategy by enabling precise control of microscale environments, real-time monitoring, dynamic perfusion, and miniaturized high-throughput analysis. More importantly, these capabilities allow the evaluation of early functional responses, concentration-dependent exposure effects, and microenvironment-regulated drug activity, thereby providing mechanistic information that is usually inaccessible in conventional endpoint assays. For example, microfluidic platforms have been developed to monitor intracellular reactive oxygen species (ROS) in real time, thereby shifting anticancer drug evaluation from static viability-based readouts toward dynamic functional assessment [116] (Figure 5A). The key advantage of this approach is that ROS perturbation can be captured as an early pharmacodynamic event preceding overt cytotoxicity, which helps distinguish primary redox-mediated drug action from secondary consequences of cell death. This is particularly relevant for TCM-derived compounds, many of which modulate oxidative stress, mitochondrial homeostasis, or stress-response pathways in a time- and dose-dependent manner.

This advantage is particularly relevant to TCM research, where the chemical composition of herbal medicines is highly complex and often characterized by multicomponent, multitarget, and synergistic effects. Although such complexity provides a rich resource for anticancer drug discovery, it also creates substantial challenges for conventional screening platforms such as multiwell plates. Traditional methods are often poorly suited to resolving combinatorial effects, require relatively large sample volumes, and are limited when testing poorly water-soluble herbal extracts or low-yield natural compounds [117]. By contrast, microfluidic systems can generate stable concentration gradients, support parallelized testing, and dramatically reduce reagent consumption, making them well suited for screening precious natural products and complex mixtures [118] (Figure 5B). Beyond these practical benefits, microfluidic gradient generation is mechanistically important because it enables systematic mapping of dose–response relationships and interaction landscapes among multiple constituents. This is highly relevant to TCM, in which efficacy frequently depends on combinatorial modulation of several targets rather than the action of a single dominant compound.

The integration of microfluidics with high-throughput screening has further accelerated the identification of active TCM-derived anticancer components. In addition to reducing sample and reagent use, these systems can improve the analysis of drug delivery behavior and cell–drug interactions, which is especially important for natural products with limited bioavailability [119]. In this context, microfluidic models that incorporate physiological barriers or tissue interfaces can help distinguish insufficient intrinsic anticancer potency from poor transport, penetration, or retention, an issue that is often obscured in standard 2D monoculture assays. For instance, Ding et al. developed a microfluidic-based print-to-screen platform that enabled parallel testing of combinatorial chemotherapy regimens while substantially reducing reagent consumption and improving screening efficiency [120] (Figure 5C). This combinatorial screening capacity is particularly meaningful for TCM studies, as many herbal formulations exert pharmacological effects through coordinated actions among multiple constituents rather than through a single dominant compound. More importantly, such platforms provide an experimental basis for quantifying synergistic, additive, or antagonistic relationships among compounds, which is much more aligned with the compatibility-based pharmacology of TCM than one-compound-at-a-time screening.

At higher analytical resolution, microfluidic technologies have also been applied to the screening of bioactive molecules from complex botanical extracts at the single-cell or molecular-target level. Single-cell microfluidic assays have been used to identify transient receptor potential (TRP) channel modulators from *Murraya* species, effectively reducing background noise and false-positive signals that commonly arise in bulk analyses [121] (Figure 5D). The mechanistic value of such single-cell approaches lies in their ability to reveal response heterogeneity and to attribute activity to direct cell-level events rather than population-averaged signals. This is important for TCM extracts, where low-abundance constituents or selective responders may be masked in conventional bulk assays. Beyond phenotypic screening, microfluidic laminar-flow chips have been combined with ultra-performance liquid chromatography (UPLC) and molecular docking for dual-target competitive screening. Using this integrated strategy, Yan et al. traced G-quadruplex-targeting antitumor activity in *Macleaya cordata* extracts to specific alkaloids, including sanguinarine and chelerythrine [122] (Figure 5E). Here, the contribution of microfluidics was not merely faster screening, but the ability to connect complex extract composition with target-selective binding behavior in a controllable and low-consumption format, thereby improving mechanistic attribution of activity within chemically complex TCM systems.

Taken together, microfluidic technology provides not only an efficient screening platform but also a mechanism-oriented experimental framework for TCM anticancer research. Its greatest strength lies in enabling dynamic, multi-parameter, and physiologically informed analysis of how complex TCM-derived interventions act on tumor cells and related microenvironments. Future studies should therefore move from single-compound verification toward formula-level and system-level investigation, which would better reflect the holistic and compatibility-based nature of TCM.

### 5.2. Microfluidic Platforms for Pharmacological Evaluation, Formulation, and Delivery of TCM-Derived Compounds

A second key question in TCM-based anti-cancer research is how to evaluate and optimize the pharmacological behavior of bioactive compounds or preparations under conditions that better reflect their extraction properties, formulation characteristics, delivery efficiency, and biological exposure. Even when bioactive TCM monomers are identified, their further pharmacological development is often limited by poor water solubility, low stability, inadequate bioavailability, and variable pharmacokinetic behavior [123]. In this context, microfluidic technology has emerged as a useful tool in TCM pharmacological research, particularly for the extraction, formulation, delivery, and efficacy evaluation of natural compounds. By enabling precise control of fluid flow, interfacial reactions, and microscale transport processes, microfluidic systems can reduce sample consumption while improving extraction efficiency, formulation consistency, and experimental reproducibility. More importantly, these systems can help connect physicochemical properties with biological behavior, thereby providing mechanistic insight into how extraction conditions, formulation characteristics, and microenvironmental exposure influence pharmacological performance.

**Figure 6:**
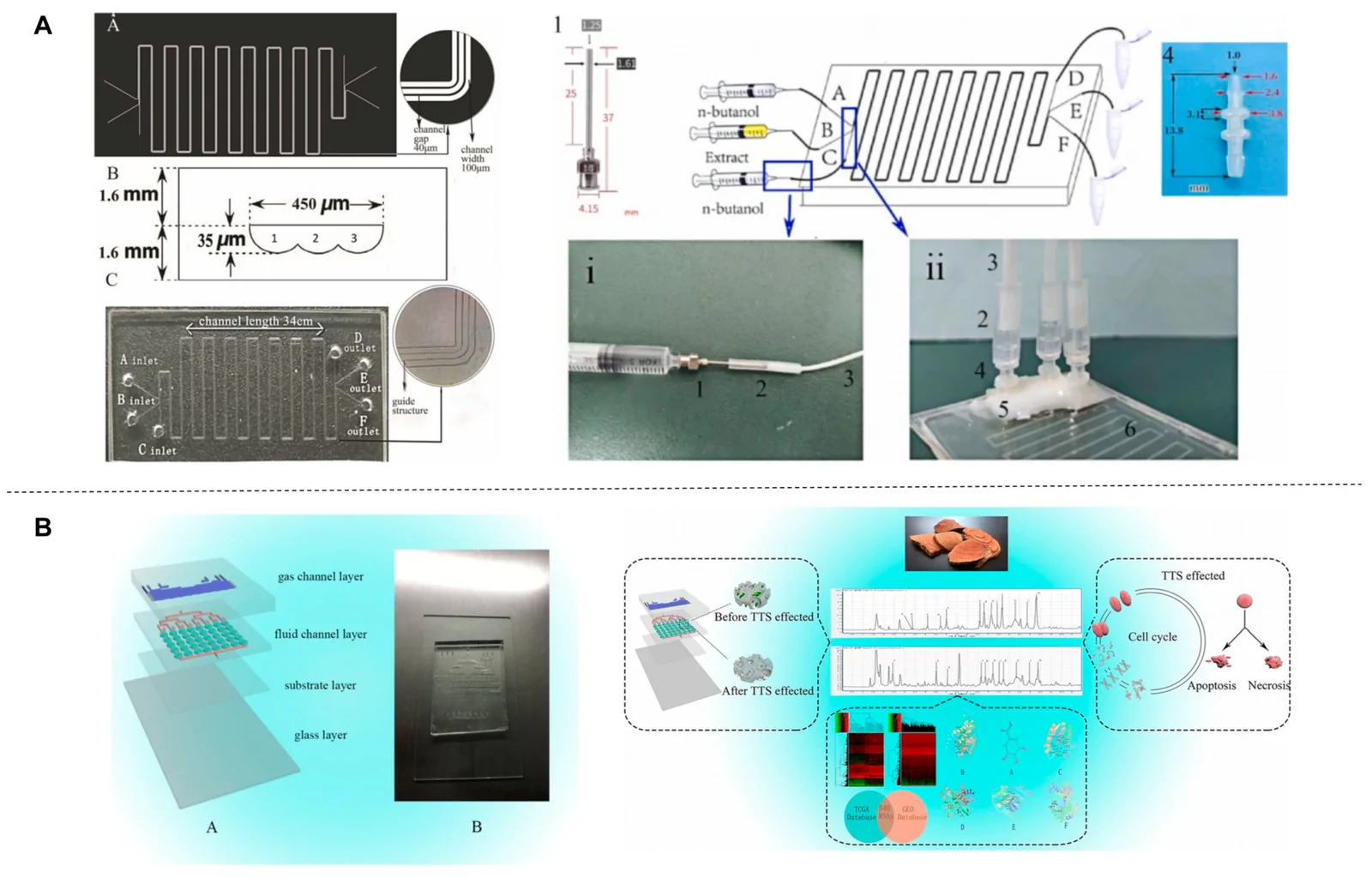
Representative microfluidic approaches in TCM pharmacological research. (**A**) An organic three-phase laminar-flow microfluidic chip for efficient, low-consumption extraction of ginsenosides from *Panax ginseng*. Reproduced with permission from Ref. [124]. (**i**) Photograph of the syringe-to-tubing connection assembly. 1: Stainless steel syringe needle; 2: Silicone tube for sealing and mechanical reinforcement; 3: Polytetrafluoroethylene (PTFE) fluidic tubing. This assembly establishes a leak-tight, low-dead-volume connection between the syringe pump and the transmission tubing, ensuring stable and pulse-free fluid delivery to the microchip. (**ii**) Photograph of the tubing-to-chip connection assembly. 2: PTFE fluidic tubing; 3: Equal-diameter straight (EDS) connector; 4: Chip inlet reservoir; 5: AB adhesive for sealing and structural fixation; 6: Glass microfluidic chip substrate. This assembly introduces the three fluid streams into their corresponding chip channels respectively, and provides reliable air-tightness to avoid bubble intrusion and flow disturbance during laminar flow establishment. Copyright 2023, Elsevier B.V. (**B**) A three-dimensional microfluidic chip used to evaluate the anti-cervical-cancer activity of Spatholobus suberectus tannins (TTS) and to validate their effects on cell viability, apoptosis, and cell-cycle regulation. Reproduced with permission from Ref. [125]. Copyright 2018, Springer Nature.

One important application of microfluidics in TCM pharmacology lies in the extraction and preparation of active compounds. Chen et al. developed an organic three-phase laminar-flow microfluidic chip that markedly reduced the consumption of samples and reagents through controlled laminar-flow design. Using this platform, they achieved an extraction efficiency of 63.1–71.6%, providing an efficient and low-consumption approach for the extraction of active ingredients from TCM materials [124] (Figure 6A). Such strategies are particularly valuable for natural products that are available only in limited quantities or require high-purity isolation for subsequent biological testing. From a mechanistic perspective, laminar-flow extraction systems also offer a controllable platform for analyzing how phase contact time, diffusion distance, and solvent partitioning affect selective enrichment of different components. This is relevant to TCM because changes in extraction conditions may alter not only yield but also the relative composition of active and inactive constituents, thereby influencing downstream pharmacological outcomes.

One aspect of this question concerns how preparation and extraction conditions influence downstream pharmacological performance. Beyond extraction, microfluidic systems also provide biomimetic platforms for functional validation of TCM monomers in physiologically relevant settings. Wang et al. investigated the antitumor activity of *Spatholobus suberectus* tannins (TTS) against cervical cancer using a three-dimensional microfluidic chip. They found that increasing TTS concentrations progressively reduced HeLa cell viability and increased apoptosis in a dose-dependent manner. In addition, TTS induced G0/G1 cell-cycle arrest, thereby inhibiting cell proliferation and supporting its therapeutic potential in cervical cancer [125] (Figure 6B). Although similar endpoints can also be measured in standard culture systems, the added value of the microfluidic 3D model lies in its ability to preserve drug diffusion constraints, nutrient gradients, and cell–matrix interactions, which can substantially alter pharmacological response. Therefore, such systems help determine whether an observed effect remains valid under more tissue-like exposure conditions rather than under oversimplified static monolayer settings.

The combination of organoid models with microenvironment-mimicking culture systems has further expanded the scope of TCM pharmacological research. Using colorectal cancer organoids, Miyazaki et al. showed that curcumin combined with andrographolide exerted enhanced antitumor effects by inducing ferroptosis and simultaneously suppressing GPX4 and FSP1, suggesting the potential of this combination as an adjuvant therapeutic strategy for colorectal cancer [126]. This finding highlights the value of biomimetic models in uncovering context-dependent mechanisms, such as ferroptosis sensitivity, that may not be fully reflected in conventional 2D assays.

Similar observations have been reported in other organoid-based tumor models. Zhao et al. treated human colorectal cancer organoids with lobeline (30 μmol/L) for 48 h and found marked inhibition of organoid growth, supporting its potential relevance in colorectal cancer treatment [127]. In another study, Liu et al. established a glioblastoma spheroid–brain organoid co-culture model to investigate antitumor mechanisms and showed that valerate significantly reduced both the number of invading glioblastoma cells and the invasion area within the brain organoid system [128]. These examples illustrate how organoid-associated microsystems can reveal mechanisms related to invasion, tissue-context-dependent growth control, and therapeutic response heterogeneity, which are often masked in conventional assays lacking preserved architecture and multicellular interactions.

Microfluidic technology is also valuable for improving the delivery of TCM-derived preparations. Hong et al. applied high-pressure microfluidic technology to prepare *Liushen Wan* lipid nanoparticles (nano-LSW). The resulting formulation exhibited rapid in vitro release, with more than 70% of the drug released within the first 6 h. Functional studies further showed that nano-LSW interfered with key tumor-microenvironment-associated factors, induced tumor-cell apoptosis, and displayed low toxicity toward normal liver cells. In vivo, nano-LSW enhanced tumor growth inhibition while causing minimal or no detectable damage to normal liver, kidney, or intestinal tissues [129]. In this case, the value of microfluidic formulation is not merely technical standardization. By producing more uniform particles with controllable release behavior, microfluidics facilitates a clearer correlation between preparation parameters and biological outcomes, which is essential for transforming empirical TCM formulations into more reproducible and mechanistically interpretable delivery systems.

Overall, microfluidic technology has shown considerable value in TCM pharmacological research by supporting extraction, formulation, delivery, and efficacy evaluation within more controllable and biologically relevant experimental settings. However, current work still focuses predominantly on individual monomers or simplified preparations, which only partially reflects the essence of TCM pharmacology. Since many TCM formulae act through coordinated absorption, metabolism, distribution, and multitarget regulation, future studies should exploit microfluidic platforms to compare whole-formula extracts with selected component combinations, to investigate herb-pair compatibility, and to analyze how formula-derived metabolites act across barriers and organs. Such a shift would allow microfluidic pharmacology to contribute more directly to the core scientific questions of TCM rather than merely validating isolated compounds.

### 5.3. Microfluidic Platforms for Toxicity Evaluation of TCM-Derived Compounds and Formulations

Drug-induced toxicity is a major cause of clinical failure and post-marketing drug withdrawal, making preclinical safety evaluation an essential component of pharmaceutical research. This issue is particularly important in TCM, where herbal medicines and formulations often contain chemically complex mixtures that may include both therapeutic constituents and potentially harmful substances. In addition, the toxicity of certain TCM materials may depend on dosage, processing methods, metabolism, and inter-organ interactions, which are difficult to capture using conventional static in vitro assays. Owing to their ability to reconstruct tissue microenvironments, enable dynamic perfusion, support real-time analysis, and model inter-organ communication, microfluidic systems have emerged as valuable tools for toxicological assessment in TCM research. Their importance lies not only in improving sensitivity or reducing sample use, but also in clarifying mechanism-dependent toxicity processes such as metabolic activation, tissue-specific susceptibility, and sequential injury across organs.

**Figure 7:**
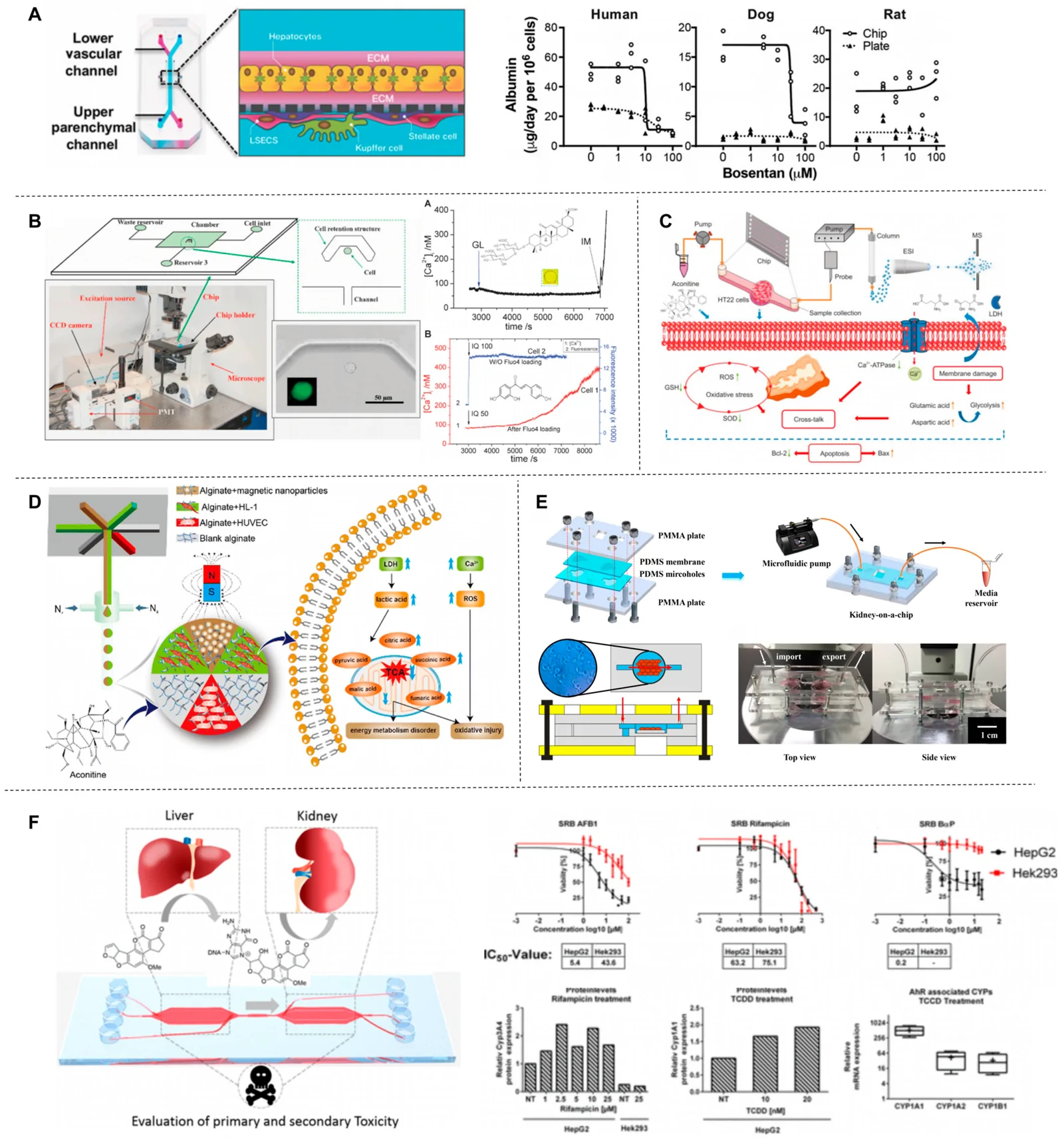
Representative microfluidic platforms for evaluating the toxicological effects of TCM-related compounds. (**A**) A cross-species Liver-Chip platform designed to recapitulate liver microenvironments in humans, rats, and dogs for predictive hepatotoxicity testing and comparative safety assessment. Reproduced with permission from Ref. [130] Copyright 2019, American Association for the Advancement of Science. (**B**) A single-cell microfluidic assay for rapid analysis of drug-induced intracellular Ca^2+^ dynamics, used to evaluate the anti-leukemia activity and cellular response of isoliquiritigenin. Reproduced with permission from Ref. [131]. Copyright 2009, The Royal Society of Chemistry. (**C**) A microfluidic chip–mass spectrometry (Chip-MS) platform used to elucidate the mechanism of aconitine-induced neurotoxicity in HT22 cells. Reproduced with permission from Ref. [132]. Copyright 2022, Elsevier B.V. (**D**) A microfluidically fabricated “heart-on-a-particle” model used to evaluate aconitine-induced cardiotoxicity in a multicompartment cardiac microenvironment. Reproduced with permission from Ref. [133]. Copyright 2024, The Royal Society of Chemistry. (**E**) A GelMA-based kidney-on-a-chip platform for nephrotoxicity evaluation of kaempferol, a major active compound of *Mentha spicata* L. Reproduced with permission from Ref. [134]. Copyright 2018, Frontiers Media S.A. (**F**) A liver–kidney-on-chip model for studying inter-organ metabolic interactions and metabolite-mediated toxicity. Reproduced with permission from Ref. [135]. Copyright 2017, American Chemical Society.

Liver-based microfluidic models are especially important because hepatic metabolism is central to both detoxification and bioactivation. Jang et al. developed a cross-species Liver-Chip platform that recapitulated key features of the liver microenvironment in humans, rats, and dogs, demonstrating its utility for predictive hepatotoxicity testing and comparative safety assessment [130] (Figure 7A). Such platforms provide a useful basis for evaluating the hepatic safety of TCM-derived compounds and, more importantly, for examining species-dependent metabolic differences that may complicate interpretation of animal toxicity data. This is particularly relevant to TCM, because discrepancies between preclinical animal studies and human responses may arise from differences in metabolic transformation rather than from the parent compounds themselves.

Microfluidics has also been applied to rapid cellular toxicity analysis at high temporal resolution. Li et al. developed a single-cell microfluidic approach for analyzing drug responses in cancer cells during TCM-related compound discovery. By monitoring intracellular calcium dynamics, this system enabled rapid detection of drug-induced cytotoxic responses without the prolonged waiting period required for conventional assays. Using this platform, the authors showed that isoliquiritigenin, a bioactive component derived from licorice, exerted anti-proliferative effects on leukemia cells by inducing sustained intracellular Ca^2+^ elevation, suggesting both pharmacological activity and a measurable toxicity-related cellular response pathway [131] (Figure 7B). Compared with conventional endpoint viability assays, this strategy provides mechanistic access to early stress signaling events and cellular heterogeneity, thereby helping to distinguish transient adaptive responses from sustained toxicity-associated perturbation.

Microfluidic platforms are particularly useful for clarifying the mechanisms underlying the toxicity of known TCM-associated toxicants. Aconitine, a representative toxic alkaloid present in processed aconite (*Fuzi*), has recognized pharmacological activity but may also cause poisoning when present at excessive levels. Using microfluidic chip–mass spectrometry (Chip-MS), Zhang et al. demonstrated that aconitine induced neurotoxicity through excessive excitatory amino acid signaling, accompanied by enhanced glycolysis, lactate accumulation, and glucose consumption, which further aggravated cellular injury. They also found that excitotoxicity led to intracellular Ca^2+^ overload, oxidative stress, mitochondrial damage, and ultimately apoptosis mediated through the Bax/Bcl-2 pathway [132] (Figure 7C). By combining single-cell capture with dynamic perfusion and real-time molecular analysis, this approach enabled temporal mapping of the cascade of aconitine-induced neurotoxic events. The principal advantage over conventional assays is that it links successive metabolic and signaling alterations into a coherent toxicity pathway rather than providing only a final injury endpoint.

In addition to neurotoxicity, microfluidic systems have been used to model organ-specific toxicity in biomimetic settings. Xu et al. used microfluidic technology to fabricate multicompartment hydrogel particles as a “heart-on-a-particle” model, in which cardiomyocytes (HL-1 cells) and HUVECs were encapsulated in separate compartments to simulate aspects of the cardiac microenvironment. Their results showed that aconitine disrupted membrane integrity, caused lactate dehydrogenase (LDH) leakage and Ca^2+^ overload, inhibited the tricarboxylic acid (TCA) cycle, induced metabolic dysfunction and oxidative stress, and ultimately triggered apoptosis [133] (Figure 7D). This study illustrates how compartmentalized microfluidic constructs can facilitate mechanistic analysis of organ-specific toxicity while preserving intercellular interactions that may modulate injury severity and pathway activation.

Microfluidic models can also be used to evaluate the safety of potentially therapeutic TCM compounds. Li et al. established a GelMA-based kidney organoid microfluidic chip to assess the nephrotoxicity of kaempferol, a major component of *Mentha spicata* L. After 12 h of treatment, cell morphology remained largely normal, with no significant evidence of damage or apoptosis at the tested concentration, suggesting an absence of notable nephrotoxicity under these conditions [134] (Figure 7E). This example highlights the utility of microfluidic kidney models for dynamic safety evaluation in a more physiologically relevant context than conventional monolayer culture, particularly when renal responses may depend on perfusion, structural organization, or metabolite exposure.

Beyond single-organ models, interconnected multi-organ chips provide additional value for investigating systemic toxicity and metabolite-mediated injury. The liver–kidney microfluidic chip developed by Theobald et al. simulated the conversion of aflatoxin B1 (AFB1) into toxic epoxide metabolites in the liver compartment via CYP3A4/1A2, followed by transport to the kidney compartment, where reduced renal cell viability was observed. In contrast, toxicity was markedly attenuated under reverse-flow conditions, supporting the conclusion that liver metabolism drove downstream nephrotoxicity [135] (Figure 7F). The same platform also enabled studies of benzo[*a*]pyrene metabolism and rifampicin–AFB1 interactions, demonstrating the broader utility of multi-organ chips for evaluating metabolism-dependent toxic responses.

From the perspective of TCM safety research, these platforms are particularly relevant because the toxicity of herbal medicines is often influenced by processing, compatibility, metabolic conversion, and tissue-specific susceptibility. Notably, this is also where the current literature is most incomplete: existing microfluidic toxicology studies have focused mainly on single toxic compounds such as aconitine, whereas formula-level detoxification mechanisms remain largely unexplored. Since traditional TCM practice often reduces toxicity through processing or herb compatibility, microfluidic models, especially interconnected organ systems, could be used in future work to determine whether specific herb combinations alter toxic metabolite formation, tissue distribution, barrier passage, or organ-specific accumulation. Such studies would provide a more mechanistic basis for evaluating classical detoxification principles and would substantially expand the relevance of microfluidics to authentic TCM safety research.

## 6. Conclusions and Perspectives

The evidence summarized in this review highlights the TME as a critical determinant of tumor progression, therapeutic response, and resistance. However, the marked spatial and temporal heterogeneity of the TME continues to limit the effectiveness of both modern anticancer therapies and TCM-based interventions. In this context, microfluidic technology has emerged as a valuable experimental platform for reconstructing complex biological microenvironments in a controllable and dynamic manner. Compared with conventional two-dimensional culture systems and animal models, microfluidic chips offer distinct advantages, including precise manipulation of cellular and biochemical gradients, low sample consumption, real-time observation, and compatibility with high-throughput analysis. These features make them particularly well suited for investigating how TCM components influence cell–cell communication, immune regulation, drug transport, stromal remodeling, and treatment resistance within the TME. More importantly, as emphasized throughout this review, the main value of microfluidic technology lies not simply in miniaturization or throughput enhancement, but in its ability to provide mechanistic insight into dynamic, multicellular, and context-dependent processes that are often difficult to capture using conventional static in vitro assays or standard animal models.

As discussed throughout this review, microfluidic systems have shown broad applicability in TCM-related cancer research, ranging from anticancer drug screening and pharmacological evaluation to toxicity assessment and mechanistic studies. By enabling in vitro reconstruction of tissue barriers, vascular-like perfusion, multicellular interactions, and organ-relevant microenvironments, these platforms help address several limitations associated with traditional models, such as species differences, limited physiological relevance, low throughput, and insufficient temporal resolution. Collectively, the studies discussed in this review indicate that microfluidic platforms can shift TCM oncology research from predominantly descriptive or phenomenological observation toward quantitative and mechanism-oriented investigation. This transition is especially important for clarifying how TCM interventions regulate tumor cell behavior, stromal interactions, immune responses, barrier transport, and toxicity-related pathways within biologically relevant microenvironments.

### 6.1. Challenges and Limitations

Despite these advances, current microfluidic TME models still face several important limitations that constrain their broader application in TCM cancer research. First, many existing platforms reproduce only selected features of the TME and do not fully capture its biological complexity, including immune heterogeneity, matrix remodeling, metabolic coupling, and systemic inter-organ interactions. As a result, their ability to reflect the full context of TCM action remains limited.

Second, many current studies still focus primarily on isolated TCM monomers or simplified extracts, whereas the therapeutic principles of TCM are often rooted in multi-component formulations, compatibility relationships, and coordinated pharmacological interactions. Future research should therefore move beyond single-compound evaluation and pay greater attention to the compatibility principles underlying TCM formulas. In particular, the traditional “Jun–Chen–Zuo–Shi” (Emperor–Minister–Adjuvant–Courier) theory may provide a valuable conceptual framework for investigating the hierarchical and synergistic actions of formula components. Rather than viewing TCM formulas as simple mixtures of active ingredients, microfluidic systems may enable researchers to examine how different components interact through concentration-dependent, time-dependent, and spatially organized mechanisms. With programmable fluidic control, future chip platforms could be designed to model sequential administration, differential distribution, barrier penetration, stromal modulation, or toxicity attenuation among formula constituents, thereby providing an experimentally tractable approach for studying the compatibility logic of TCM.

In addition, limited standardization and reproducibility remain practical obstacles in the field. Variations in chip architecture, material selection, flow conditions, matrix composition, cell sources, and herbal preparation methods can all influence experimental outcomes, making cross-study comparison difficult. Long-term co-culture stability also remains challenging, particularly when multicellular systems involving tumor, stromal, endothelial, and immune components are required.

Another important limitation is the current lack of integrated, high-resolution, real-time monitoring in many microfluidic studies. Although existing platforms have already improved observation of dynamic biological responses, future systems would benefit from coupling with more advanced online analytical tools. The integration of fluorescence-based sensing, electrochemical detection, microdialysis sampling, liquid chromatography–mass spectrometry, and other multimodal readout technologies may allow continuous monitoring of pH, oxygen, metabolites, cytokines, and drug distribution within chip-based models. Such developments would further strengthen the ability of microfluidics to capture the dynamic and multi-target characteristics of TCM action.

Moreover, metabolism and systemic pharmacokinetics are still insufficiently represented in many current models. Because the efficacy and toxicity of TCM may depend on metabolic transformation and inter-organ interactions, single-organ or localized tumor chips cannot fully recapitulate these processes. In addition, the translational relevance of many studies remains limited by the insufficient use of patient-derived materials and the lack of standardized validation against in vivo or clinical findings.

### 6.2. Future Perspectives

Looking ahead, the convergence of microfluidics with organoid technology, single-cell analysis, spatial omics, biosensing, and artificial intelligence is likely to further expand its value in TCM oncology research. One promising direction is the development of multidimensional datasets linking specific TCM component combinations with TME remodeling phenotypes, molecular signaling changes, and therapeutic outcomes. Another important direction is the construction of patient-derived or patient-specific chip models incorporating autologous immune cells, tumor organoids, and stromal components, which may support personalized evaluation of TCM-based combination strategies and improve prediction of clinical response. At the same time, future efforts should also focus on developing more standardized and biologically integrated chip platforms, strengthening long-term multicellular maintenance, and expanding toward metabolism-related and multi-organ models, so that the major limitations of current systems can be more effectively addressed.

Overall, the holistic philosophy of TCM and the precise modeling capacity of microfluidic technology should not be viewed as contradictory but rather as potentially complementary. TCM emphasizes system-level regulation and dynamic balance, whereas microfluidics provides a means to dissect and quantify the biological processes underlying such effects in defined experimental settings. As microfluidic technology continues to integrate with advanced analytical and bioengineering approaches, it is expected to play an increasingly important role in clarifying the mechanisms of TCM, optimizing formula design, improving safety evaluation, and accelerating translational research.

In summary, this review suggests that the future development of this field should prioritize more faithful reconstruction of TME complexity, stronger incorporation of authentic TCM formula principles, improved standardization and real-time analysis, and broader use of patient-derived and multi-organ platforms. Ultimately, such interdisciplinary integration may provide a more rigorous and efficient route for advancing TCM-based anticancer therapies from laboratory investigation toward clinical application.

## Figures and Tables

**Table 1:** Drugs targeting tumor microenvironment.

Representative Drugs	Strategy	Target/Mechanism	Cancer Types	Status	References
Pembrolizumab	Immune checkpoint blockade	Blocks PD-1/PD-L1 interaction and restores T-cell activity	Melanoma, NSCLC, gastric cancer, etc.	Approved	[36]
Atezolizumab	Immune checkpoint blockade	Targets PD-L1 to restore antitumor immune responses	NSCLC, urothelial carcinoma, etc.	Approved	[37]
Ipilimumab	Immune checkpoint blockade	Blocks CTLA-4 and enhances T-cell activation	Melanoma and other tumors	Approved	[38]
Bevacizumab	Anti-angiogenic therapy	Neutralizes VEGF and suppresses abnormal angiogenesis	Ovarian cancer and other solid tumors	Approved	[39]
Cabozantinib	Multi-target TME modulation	Inhibits VEGFR1–3, MET, and AXL, affecting angiogenesis and tumor–stroma signaling	Advanced hepatocellular carcinoma and other tumors	Approved	[40]
Pexidartinib	TAM-targeted therapy	CSF1R inhibitor; reduces macrophage recruitment and protumor polarization	TGCT, solid tumors	Approved/clinical use in selected indications	[41]
Vimseltinib	TAM-targeted therapy	CSF1R inhibitor; modulates TAM recruitment and polarization	TGCT	Clinical development	[42]
Epacadostat	Immunometabolic modulation	IDO1 inhibitor; reduces tryptophan catabolism-mediated immunosuppression	Melanoma	Clinical development	[43]
S-3304	Matrix remodeling inhibition	Inhibits MMPs involved in invasion and ECM remodeling	Advanced solid tumors	Clinical development	[44]
Tocilizumab	Cytokine signaling modulation	Inhibits IL-6 signaling and alleviates inflammatory/immunosuppressive conditions	Advanced NSCLC	Clinical development	[45]

**Table 2:** Representative Chinese herbal medicines involved in tumor microenvironment regulation.

Chinese Medicine/Herbal Source	Active Component or Formulation	Cancer Type/Model	Major TME-Related Mechanism	References
*Astragali Radix*	Astragalus Polysaccharides (APS)	Breast cancer; macrophage/DC-related cell and mouse models	Activates TLR4/MyD88 signaling; promotes M1 macrophage polarization and dendritic cell maturation	[55]
*Astragali Radix*	Astragalus Polysaccharides (PG2)	Lung cancer; cell and mouse models	Regulates macrophage polarization, inhibits NF-κB/CD31-related signaling, and promotes dendritic cell maturation	[57]
*Astragali Radix*	*A. membranaceus* polysaccharides (AMP)	Immune cell models and mice	Activates TLR4-related signaling; enhances antigen presentation and T-cell activation	[58]
*Ginseng Radix et Rhizoma*	Ginseng Polysaccharides (GPs)	Lung cancer; tumor-bearing mouse models	Remodels gut microbiota, inhibits IDO-associated immunosuppression, and enhances T-cell function	[56]
*Ginseng Radix et Rhizoma*	Ginsenoside Rh2 (G-Rh2)	Lung cancer; cell and mouse models	Promotes M1 polarization and suppresses VEGF/MMP-2/MMP-9-related invasion and angiogenesis	[59]
*Tripterygii Wilfordii Radix*	Celastrol Nanoemulsion (CEL NE)	Melanoma; cell and mouse models	Induces immunogenic cell death, downregulates PD-L1, and remodels the immunosuppressive TME	[60]
*Tripterygii Wilfordii Radix*	Celastrol (CSL)	Myeloma-related cell and xenograft models	Inhibits NF-κB signaling and inflammatory mediators, improving the tumor-promoting inflammatory milieu	[61]
*Scutellariae Radix*	Baicalein	Colorectal cancer; cell and animal models	Inhibits angiogenesis via the TLR4/HIF-1β/VEGF pathway	[62]
*Smilacis Glabrae Rhizoma*	Aiphanol	Colon cancer; endothelial and animal models	Dual inhibition of VEGFR2 and COX2 suppresses tumor angiogenesis	[63]
*Silybi Marianae Fructus*	ARm@SNP (silybin-loaded biomimetic nanoparticles)	Breast cancer; cell and mouse models	Targets CAFs and disrupts stromal and immunosuppressive barriers	[64]
*Bruceae Fructus*	Bruceine A	Pancreatic cancer; cell and mouse models	Inhibits glycolytic reprogramming through PFKFB4/GSK3β signaling	[65]
*Ganoderma*	*Ganoderma tsugae* ethanol extract (GTEE)	Prostate cancer; cell models	Inhibits the SREBP-1/AR axis and lipid metabolic reprogramming	[66]

**Table 3:** Comparison of cellular, animal, and microfluidic models for tumor microenvironment simulation.

Model	Advantages	Disadvantages	Advantages for TME Simulation	References
Cellular Model	Simple operation, low cost, high compatibility; Allows precise single-variable control and uniform cell distribution	Lacks 3D spatial architecture and microenvironmental complexity; Limited physiological relevance; Poor prediction of in vivo drug responses	Suitable for single-factor mechanistic studies; highly compatible with standard molecular and cellular assays	[85,86]
Animal Model	Provides a complete living systemic environment; Enables long-term investigation of disease progression; Offers translational relevance	Significant interspecies differences may affect results and drug efficacy evaluation; High cost, ethical concerns and long experimental cycle	Can simulate an intact living TME, but the structure and immune context differ from those of human tumors	[87]
Microfluidic Model	Highly biomimetic; Integrates 3D culture with real-time imaging; Allows precise and independent control of multiple physiological variables; Reduces reliance on animal testing	Requires high initial investment and specialized technical expertise; Current models are still limited in replicating the absolute full-body complexity of a living organism	Effectively reconstructs key TME components; enables isolation and control of specific cellular and physical variables; supports dynamic and real-time observation	[81]

## Data Availability

The data presented in this study are available on request from the corresponding author.

## Abbreviations

2D	Two-Dimensional
3D	Three-Dimensional
3D-CMOM	Three-Dimensional Co-culture Multiorgan Microfluidic
AFB1	Aflatoxin B1
APS	Astragalus Polysaccharides
ARm@SNP	Anisamide-modified red blood cell membrane-coated silybin-loaded nanoparticles
AQP2	Aquaporin 2
BBB	Blood–Brain Barrier
B7-H3	B7 Homolog 3
CAFs	Cancer-Associated Fibroblasts
CAR-T	Chimeric Antigen Receptor T-cell
Chip-MS	Microfluidic Chip–Mass Spectrometry
COX2	Cyclooxygenase-2
CSF1R	Colony-Stimulating Factor 1 Receptor
CTLA-4	Cytotoxic T-Lymphocyte-Associated Protein 4
CXCL1	C-X-C Motif Chemokine Ligand 1
DCIS	Ductal Carcinoma In Situ
ECM	Extracellular Matrix
EGF	Epidermal Growth Factor
EMT	Epithelial–Mesenchymal Transition
ESCC	Esophageal Squamous Cell Carcinoma
FGFs	Fibroblast Growth Factors
FSP1	Ferroptosis Suppressor Protein 1
GPX4	Glutathione Peroxidase 4
GPs	Ginseng Polysaccharides
G-Rh2	Ginsenoside Rh2
GSK3β	Glycogen Synthase Kinase 3 Beta
HGF	Hepatocyte Growth Factor
HIF	Hypoxia-Inducible Factor
hiPSCs	Human Induced Pluripotent Stem Cells
hPSCs	Human Pluripotent Stem Cells
hTPV	Human Tumor–Perivascular
IDO	Indoleamine 2,3-Dioxygenase
IL-6	Interleukin-6
LDH	Lactate Dehydrogenase
Lgr5	Leucine-Rich Repeat-Containing G Protein-Coupled Receptor 5
MMPs	Matrix Metalloproteinases
μFCA	Microfluidic Cell Array
nano-LSW	Nanoparticle-Encapsulated Liushenwan
NK cells	Natural Killer cells
OOC	Organ-on-a-Chip
PD-1	Programmed Cell Death Protein 1
PD-L1	Programmed Death-Ligand 1
PDMS	Polydimethylsiloxane
PFKFB4	6-Phosphofructo-2-Kinase/Fructose-2,6-Biphosphatase 4
PSMA	Prostate-Specific Membrane Antigen
ROS	Reactive Oxygen Species
SREBP-1	Sterol Regulatory Element-Binding Protein 1
TCA	Tricarboxylic Acid
TCM	Traditional Chinese Medicine
TAMs	Tumor-Associated Macrophages
TME	Tumor Microenvironment
TLR4	Toll-Like Receptor 4
Tregs	Regulatory T cells
TRP	Transient Receptor Potential
TTS	Tannins of Spatholobus suberectus
UPLC	Ultra-Performance Liquid Chromatography
VEGF	Vascular Endothelial Growth Factor
VEGFR2	Vascular Endothelial Growth Factor Receptor 2
